# Dendritic Hydrogels Induce Immune Modulation in Human Keratinocytes and Effectively Eradicate Bacterial Pathogens

**DOI:** 10.1021/jacs.1c07492

**Published:** 2021-10-12

**Authors:** Yanmiao Fan, Soumitra Mohanty, Yuning Zhang, Mads Lüchow, Liguo Qin, Lisa Fortuin, Annelie Brauner, Michael Malkoch

**Affiliations:** † School of Chemical Science and Engineering, Fiber and Polymer Technology, KTH Royal Institute of Technology, Teknikringen 56-58, SE-100 44 Stockholm, Sweden; ‡ Department of Microbiology, Tumor and Cell Biology, Karolinska Institutet, SE-17165 Stockholm, Sweden; § Division of Clinical Microbiology, Karolinska University Hospital, Solna, Stockholm SE-17176, Sweden; ∥ Institute of Design Science and Basic Components, Xían Jiaotong University, 710049 Xían, P. R. China

## Abstract

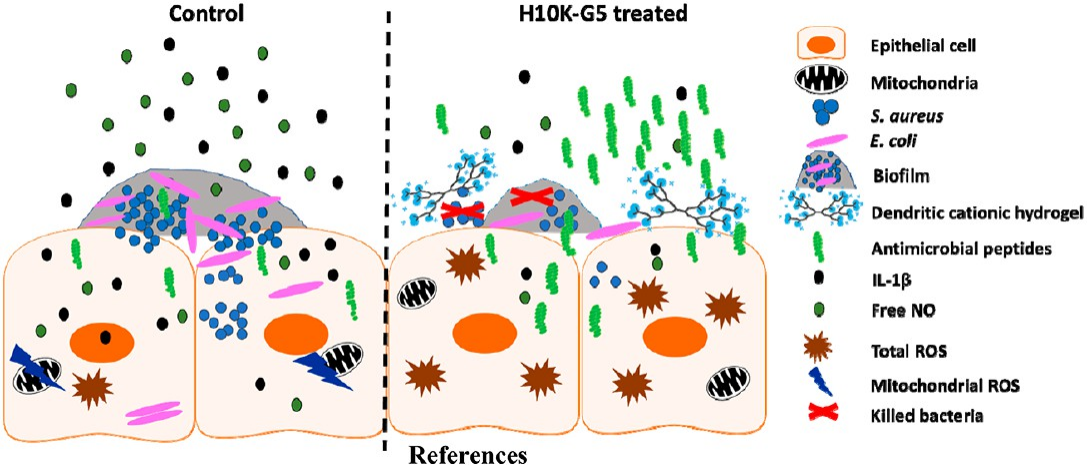

Infections caused by antibiotic-resistant bacteria are globally a major threat, leading to high mortality rates and increased economic burden. Novel treatment strategies are therefore urgently needed by healthcare providers to protect people. Biomaterials that have inherent antibacterial properties and do not require the use of antibiotics present an attractive and feasible avenue to achieve this goal. Herein, we demonstrate the effect of a new class of cationic hydrogels based on amino-functional hyperbranched dendritic–linear–dendritic copolymers (HBDLDs) exhibiting excellent antimicrobial activity toward a wide range of clinical Gram-positive and Gram-negative bacteria, including drug-resistant strains isolated from wounds. Intriguingly, the hydrogels can induce the expression of the antimicrobial peptides RNase 7 and psoriasin, promoting host-mediated bacterial killing in human keratinocytes (HaCaT). Moreover, treatment with the hydrogels decreased the proinflammatory cytokine IL-1β, reactive nitrogen species (NO), and mitochondrial reactive oxygen species (ROS) in *S. aureus*-infected HaCaT cells, conjunctively resulting in reduced inflammation.

## Introduction

The overuse and misuse of conventional antibiotics,^1,2^ together with the accumulation of nondegradable antibiotics, contribute to the alarming evolution of resistant bacteria.^3^ Emerging multidrug-resistant (MDR) bacteria escalate the situation as there are few or no effective antibiotics left to use against these bacteria.^4,5^ It is therefore critical that we develop alternative treatment strategies to combat infections caused by such bacteria.^6^

To fight invading microorganisms, the body is equipped with antimicrobial peptides (AMPs). These endogenously expressed host defense molecules protect us from invading pathogens. AMPs including cathelicidin,^7^ β defensin-2, RNase7,^8,9^ and psoriasin^10^ serve as the first-line defense to protect the keratinocytes from infection. Most AMPs are small and cationic in nature with weak binding ability to eukaryotic cells. However, they strongly bind to the negatively charged bacterial membrane, leading to cell death by disrupting the bacterial surface. Resistance to antimicrobial peptides seldom develops, and AMPs have shown great potential not only against bacterial infections^11^ but also as a promising means to prevent biofilm formation and as anticancer agents.^12^

Antibacterial polymers are good alternatives to traditional small molecule antibiotics when considering their high efficacy as well as low toxicity and resistance, combined with their limited environmental impact.^13−15^ Chin and coauthors^16^ synthesized guanidinium-functionalized polycarbonates that do not lead to drug resistance even after repeated exposure of the polymers to bacteria for 30 times, and the biodegradability of the polymers further reduced the accumulation of the polymers in that environment. Cationic macromolecules such as antimicrobial peptides,^17,18^ cationic peptidopolysaccharides,^19^ cationic dendrimers,^20,21^ and their hydrogels^22,23^ have shown great promise as broad-spectrum antibacterial materials. The primary bacterial membrane-disrupting mechanism of the cationic materials not only leads to their efficient bacterial killing ability but also reduces the potential to induce drug resistance.^24,25^ Cationic antimicrobial peptides and dendrimers, however, display inherent cytotoxicity toward diverse primary cells at high concentrations^26^ or at high generations of the dendrimers^27^ that limit their therapeutic applications.^28,29^ To reduce the cytotoxicity of the materials, synthetic cationic polymers that mimic AMPs are attracting increasing attention due to properties which can be tuned to meet the requirements of highly advanced antibacterial materials that can mask the cytotoxicity of the active unit.^30^ Hyperbranched polymers with highly branched three-dimensional (3D) architectures possess unique physicochemical features such as abundant functional groups, intramolecular cavities, low viscosities, and high solubility as well as the possibility of large-scale production via facile methods.^31,32^ Cationic hyperbranched polymers that mimic AMPs, in particular, make excellent antibacterial materials^33^ and hereby form the basis of this work.

Herein, a library of cationic dendritic hydrogels with inherent antibacterial properties were developed with promising results for treatment of skin wound infections. The hydrogels exhibit broad-spectrum antibacterial activity as well as good biocompatibility and degradability in a suitable time frame. Most importantly, the cationic hydrogels can induce innate immune responses in HaCaT, keratinocytes, leading to efficient bacterial killing both extracellularly and intracellularly together with reduced inflammation. The present study demonstrates that functional hydrogels can be carefully designed to achieve tuned and optimal therapeutic effects.

## Results and Discussion

### Synthesis and Characterization of Amino-Functional HBDLDs and Hydrogels

**Figure 1:**
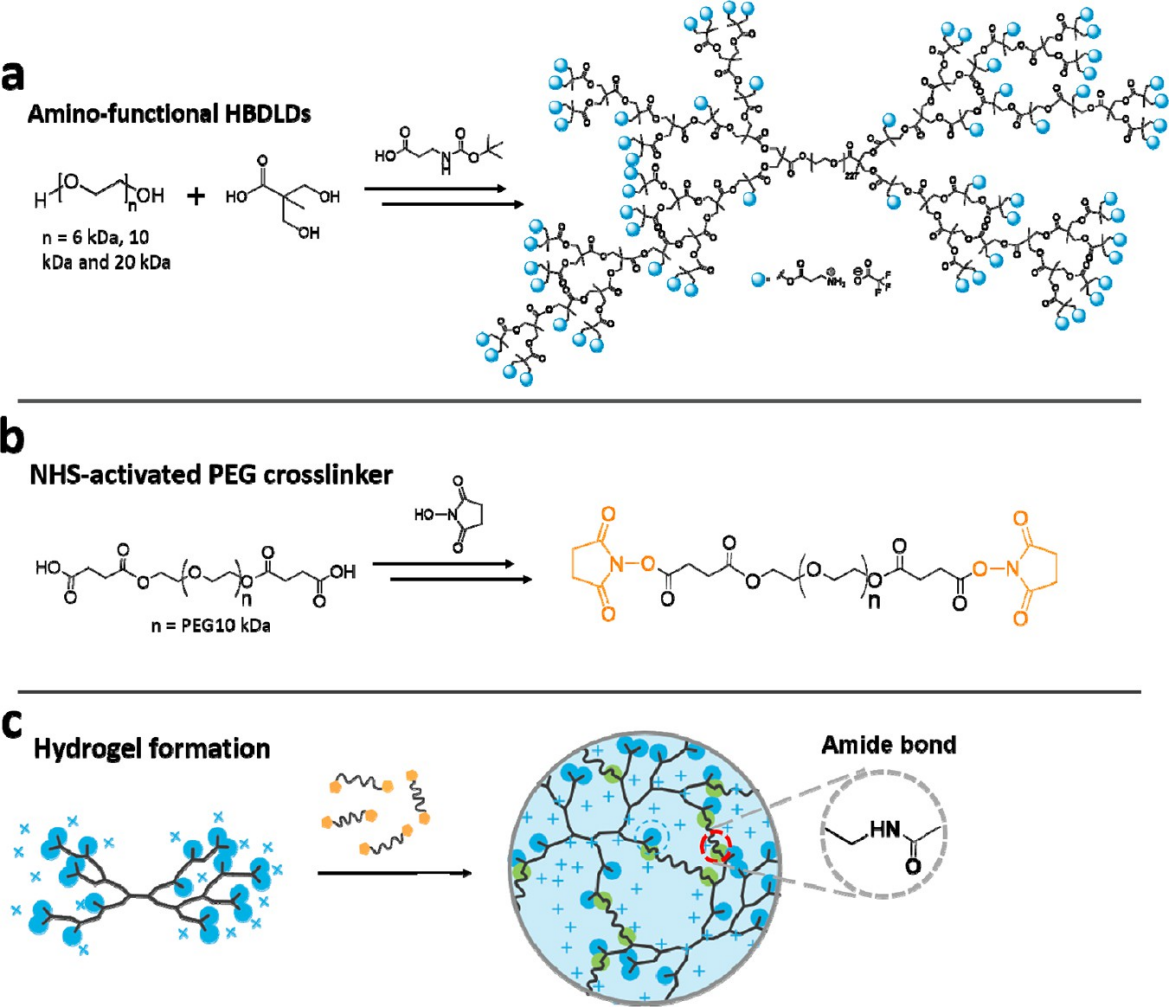
Schematic diagram describing the synthesis of (a) amino-functional HBDLDs, (b) NHS-activated PEG cross-linker, and (c) and amino-functional hydrogels.

A new class of amino-functional hyperbranched dendritic–linear–dendritic copolymers (HBDLDs) based on poly(ethylene glycol) (PEG) and 2,2-bis(hydroxymethyl)propionic acid (bis-MPA) were synthesized through the conventional pseudo-polycondensation reaction.^22,34^ Hydroxy-terminated HBDLDs were modified with boc-protected β-alanine by using fluoride-promoted esterification (FPE) chemistry,^35^ followed by the removal of boc groups by using trifluoroacetic acid (TFA). The amino-functional HBDLDs are amphiphilic copolymers, composed of a linear hydrophilic PEG core and dendritic hydrophobic components (Figure 1a) of fifth generation (G5; 64 amines per molecule) or sixth generation (G6; 128 amines per molecule). *N*-Hydroxysuccinimide (NHS) was used to activate the carboxyl-functional PEG (10 kDa) cross-linker (Figure 1b). Because of the rapid reaction rate of the NHS-mediated amidation reaction, the hydrogels can easily be formed in aqueous solution by simple mixing of the amino-terminated HBDLDs and the cross-linkers (Figure 1c). Eight amino groups out of 64 or 128 amino groups per molecule were used for the cross-linking, leaving sufficient pendant amine groups that could provide the hydrogels with antibacterial activities.

**Figure 2:**
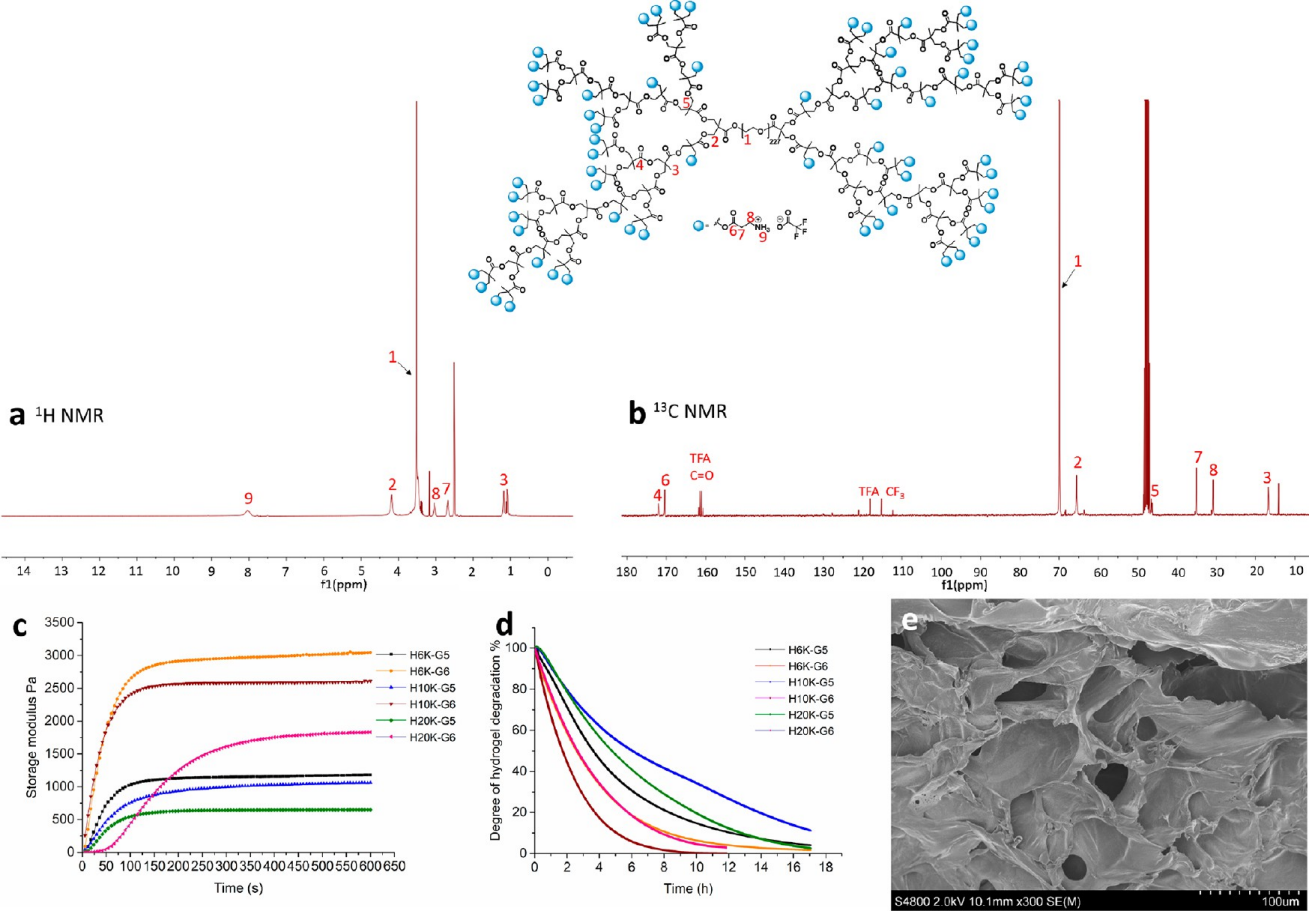
Characterization of the amino-functional HBDLDs and hydrogels. (a) ^1^H NMR spectroscopy spectrum of PEG10K-G5-NH_3_^+^ in DMSO-*d*_6_. (b) ^13^C NMR spectroscopy spectrum of PEG10K-G5-NH_3_^+^ in MeOD. (c) Gelation process monitored by rheology at room temperature; time sweeps were performed by using strain (ϒ) = 1% and frequency (ω) = 1 Hz. (d) Normalized storage modulus as a function of time during hydrogel degradation measured by a time sweep in the rheometer with the sample submerged in PBS (pH 7.4) buffer at 37 °C. (e) SEM image of the hydrogel (H10K-G5) showing porous structure.

The HBDLDs and cross-linkers were characterized via NMR spectroscopy (Supporting Information). NMR spectroscopy was used to confirm that the HBDLDs had reached completion by the absence of free carboxylic groups emanating from bis-MPA monomer or their oligomers as well as comparing the theoretical and calculated ratios of protons from the methyl groups of bis-MPA, methylene groups of PEG, and the internal methylene group of bis-MPA (Figure S1). The full substitution of the hydroxy groups with boc-protected β-alanine was confirmed by ^13^C NMR spectroscopy; after the functionalization of boc-protected β-alanine at the terminal of HBDLDs, the peak of bis-MPA at the terminal (50.2 ppm, (−COO–**C**–((CH_2_–OH)_2_) totally disappeared, and peak f (at 36.08 ppm) and peak g (at 34.38 ppm) corresponding to β-alanine appeared in the ^13^C NMR spectroscopy (Figure S2). Figure 2 showed the ^1^H NMR (Figure 2a) and ^13^C NMR (Figure 2b) spectra of amino-functional HBDLDs. Peak 9 in Figure 2a represented the amine groups of β-alanine. The β-alanine peaks in ^13^C NMR (Figure 2b) were presented at 35.02 ppm (peak 7) and 30.83 ppm (peak 8), and together with ^19^F NMR (Figure S3) spectra, the functionalization of HBDLDs with β-alanine had occurred efficiently. The hydrogels were named based on the generation and PEG length (e.g., H10K-G5 represents the hydrogel formed from PEG10K-G5-NH_3_^+^). The hydrogel formation process and their mechanical properties were analyzed by using rheology (Figure 2c). The precursor solution was placed in the rheometer and premixed for 2 s before the measurement. The storage moduli of the hydrogels increased rapidly and reached a stable plateau within minutes, indicating that hydrogel gelation is very efficient and fast. The storage moduli of the hydrogels ranged from 638 to 2969 Pa. Different from most traditional wound dressing materials, these hydrogels can be degraded within 24 h (Figure 2d) due to the rapid hydrolysis of ester bonds of the dendritic component at physiological pH of 7.4. As potential antibacterial wound dressing materials, it is therefore no need to change the wound dressing, which could be a cause of secondary damage to the wounds.^36^ The degradability of the hydrogels is also beneficial in minimizing the probability of developing drug-resistant strains because the antibacterial materials do not have sufficient time to accumulate and cause resistance in the environment.^37^ Scanning electron microscopy (SEM) images of the hydrogels are shown in Figure 2e and Figure S4 where the porous structure of the hydrogels is clearly observed. Porosity is known to be highly beneficial in accelerating wound healing, as it improves cell migration as well as water and oxygen transfer.^38^

**Figure 3:**
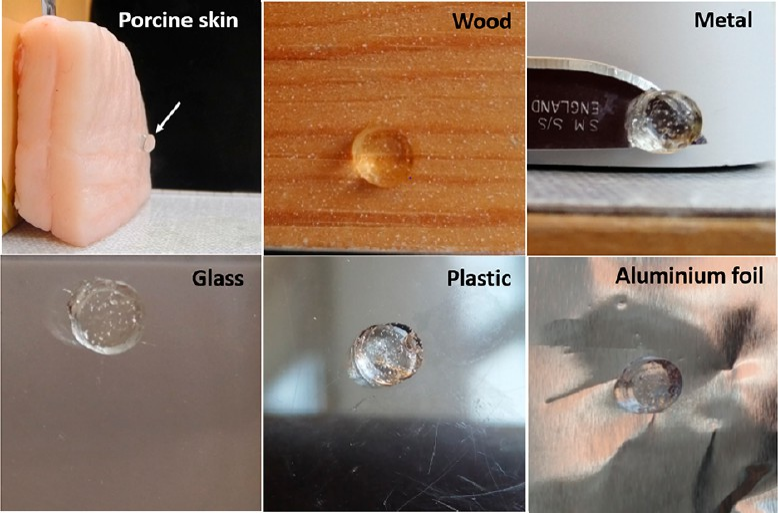
Adhesive property of the cationic hydrogels on different surfaces.

The intrinsically adhesive property of the wound dressing materials is highly advantageous in the healing process.^39^ Proper adhesion of the material to the skin surface would ensure minimal damage to the wound and surrounding skin by avoiding extra fixation and secondary damage when removing the fixation.^36^ The cationic hydrogels can adhere to raw porcine skin and various other surfaces such as wood, metal, glass, plastic, and aluminum foil (Figure 3), confirming the adhesive property and their potential use as antibacterial coating materials. The adhesive property of the hydrogels is probably due to the presence of amine groups.^40^ In addition, the adhesive force of the hydrogel can withstand intense shaking and bending (Video S1), indicating that the hydrogel can be applied on the skin without the need of extra fixation.

### Antibacterial Study of the Amino-Functional HBDLDs

The antibacterial properties of the amino-functional HBDLDs, hydroxy-terminated HBDLDs, and β-alanine were investigated by using minimum inhibitory concentration (MIC) and minimum bactericidal concentration (MBC) assays. MIC is the lowest concentration of an agent that will inhibit the visible growth of a microorganism, while MBC is the lowest concentration of a chemical needed to kill a microorganism. The antibacterial property of the cationic polymers is related to the density of amino groups, with respect to both configuration and the molecular weight of the polymers.^22,41^ Hydroxy-terminated HBDLDs and β-alanine showed no obvious antibacterial activity, with MIC values above 2000 μg mL^–1^ (Table S1), while the amino-functional HBDLDs showed excellent broad-spectrum antibacterial properties against wound-related bacteria including *Staphylococcus aureus* (*S. aureus*), methicillin-resistant *S. aureus* (MRSA), Group A streptococcus (GAS), *Pseudomonas aeruginosa* (*P. aeruginosa*), and *Escherichia coli* (*E. coli*) (Table 1). Some of the amino-functional HBDLDs inhibited the growth of *P. aeruginosa* and *E. coli* at low concentrations in the nanomolar range. For example, 6K-G6 has a MIC value of 15.6 μg mL^–1^ toward *P. aeruginosa*, with the molar concentration of 0.35 μM. MIC or MBC values of 6K-G6 and 10K-G6 toward *E. coli* 208 are 15.6 μg mL^–1^ (0.35 μM) and 31.3 μg mL^–1^ (0.65 μM), respectively. 6K-G6 exhibits the best efficacy toward most tested bacteria primarily due to its shorter PEG lengths (lower molecular weight compared with PEG10K and PEG20K) and more concentrated amino groups at the higher generation (G6). Furthermore, most MIC values are similar to MBC values, evident of the remarkable bactericidal effects of the amino-functional HBDLDs.

**Table 1:** MIC and MBC Values of the Amino-Functional HBDLDs^a^

		bacteria													
		*E. coli* 178		*E. coli* 208		*S. aureus* 2569		*S. aureus* 7920		MRSA		*P. aeruginosa* 22644		GAS	
	polymers	μg/mL	μM	μg/mL	μM	μg/mL	μM	μg/mL	μM	μg/mL	μM	μg/mL	μM	μg/mL	μM
MIC	6K-G5	15.6	0.63	15.6	0.63	125	5.02	125	5.02	125	5.02	62.5	2.51	125	5.02
	6K-G6	31.3	0.71	15.6	0.35	62.5	1.42	125	2.84	125	2.84	15.6	0.35	125	2.84
	10K-G5	62.5	2.16	62.5	2.16	250	8.64	125	4.32	125	4.32	62.5	2.16	125	4.32
	10K-G6	31.3	0.65	31.3	0.65	62.5	1.30	125	2.60	125	2.60	31.3	0.65	125	2.60
	20K-G5	125	3.21	250	6.42	125	3.21	125	3.21			125	3.21		
	20K-G6	125	2.14	125	2.14	62.5	1.07	125	1.07	250	4.28	62.5	1.07	250	4.28
MBC	6K-G5	15.6	0.63	31.3	1.76	125	5.02	125	5.02	125	5.02	62.5	2.51	125	5.02
	6K-G6	31.3	0.71	15.6	0.35	62.5	1.42	125	2.84	125	2.84	15.6	0.35	125	2.84
	10K-G5	62.5	2.16	62.5	2.16	250	8.64	125	4.32	125	4.32	62.5	2.16	125	4.32
	10K-G6	62.5	1.30	31.3	0.65	62.5	1.30	125	2.60	125	2.60	31.3	0.65	125	2.60
	20K-G5	125	3.21	250	6.42	250	6.42	250	6.42			125	3.21		
	20K-G6	125	2.14	125	2.14	125	2.14	125	2.14	250	4.28	62.5	1.07	250	4.28

a *S. aureus* 2569, *S. aureus* 7920, MRSA, *P. aeruginosa* 22644, and GAS (group A streptococcus) are all isolated from wounds. The amino-functional polymers were abbreviated, e.g., 6K-G5 represents PEG6K-G5-NH_3_^+^ with PEG length of 6K and the fifth generation.

**Figure 4:**
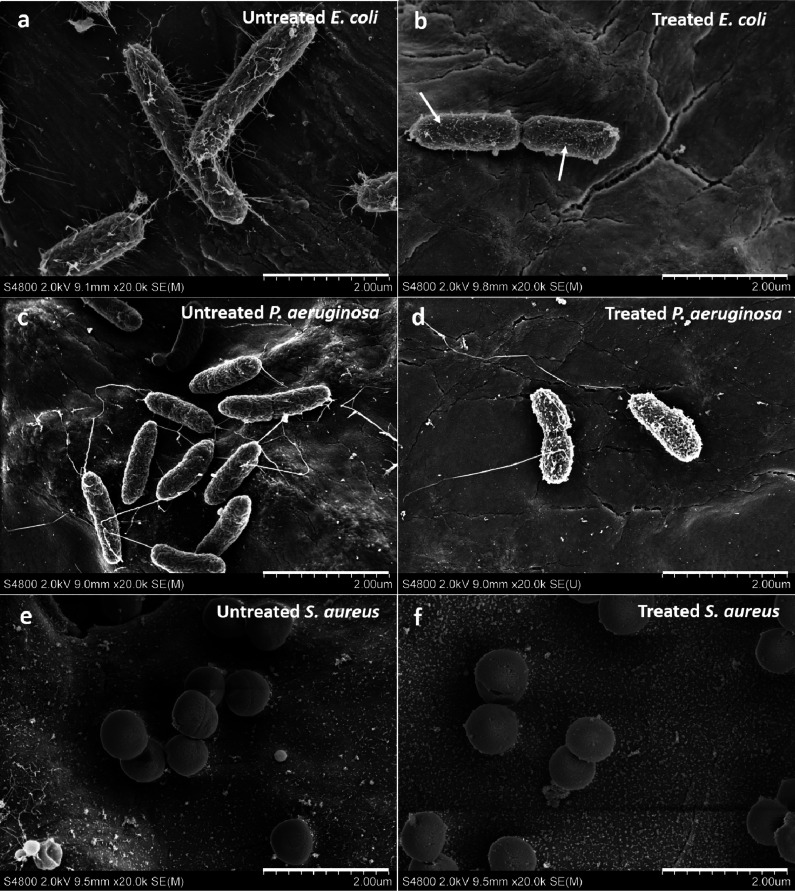
SEM images showing the morphological changes of bacteria with and without treatment of 250 μg mL^–1^ PEG10K-G5-NH_3_^+^ for 6 h at 37 °C. (a) Untreated *E. coli* 178 with visible fimbriae. (b) Treated *E. coli* 178 with damaged cell surfaces and reduced number of fimbriae. (c) Untreated *P. aeruginosa* 22644. (d) Treated *P. aeruginosa* 22644 with porosity on the cell surfaces. (e) Untreated *S. aureus* 7920. (f) Treated *S. aureus* 7920 with damaged cell surfaces.

The effects of the amino-functional HBDLDs on the morphology of *E. coli*, *P. aeruginosa*, and *S. aureus* were investigated by using SEM. As seen from Figure 4, distinct changes occurred in all strains of bacteria that were treated with 250 μg mL^–1^ of PEG10K-G5-NH_3_^+^ and incubated for 6 h at 37 °C. Not only did the amino-functional HBDLDs damage the cell membrane of *E. coli*, but the materials also had a large impact on *E. coli* fimbriae (Figure 4a,b), forming particles on the surface of the bacteria (arrows, Figure 4b). *E. coli* fimbriae play an important role in the attachment to the host cell surface as the first step in the bacterial infection^42^ and are a prerequisite to the formation of biofilms^43^ and evading extracellular antibiotics.^44^ The results suggest that the amino-functional HBDLDs exhibit great potential in preventing the formation of *E. coli* biofilms. Compared with untreated *P. aeruginosa* cells (Figure 4c), treated bacteria exhibited small holes in the cell membranes (Figure 4d). Obvious shrinkage occurred compared with the untreated *P. aeruginosa*, indicating leakage of the cell content after incubation with the PEG10K-G5-NH_3_^+^. Treated *S. aureus* exhibited clear indications of damaged cell membranes (Figure 4f) while untreated *S. aureus* cells displayed the typical smooth, spherical shapes (Figure 4e). The amino-functional HBDLDs imparted similar disrupting effects on the anionic bacterial membranes as compared to other documented cationic polymers^45^ and were able to target *E. coli* fimbriae. In addition, there was a marked decrease in the number of attached *E. coli* and *P. aeruginosa* after treatment with PEG10K-G5-NH_3_^+^ (Figure S5).

### Cytotoxicity Evaluation of the Amino-Functional HBDLDs and Hydrogels

**Figure 5:**
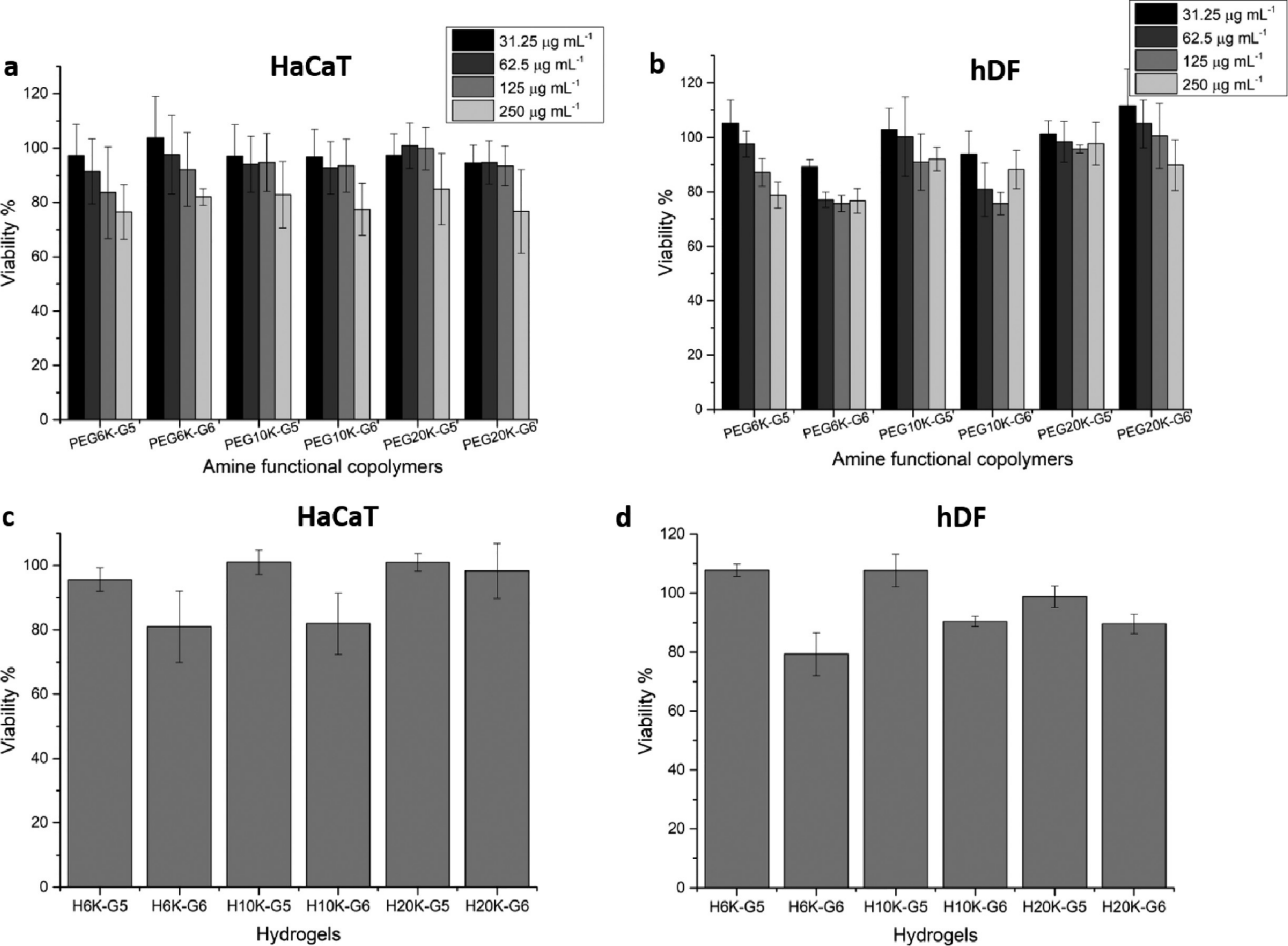
Cytotoxicity evaluation of the amino-functional HBDLDs and hydrogels. (a) The amino-functional HBDLDs toward HaCaT cells determined by XTT assay and (b) hDF cells using the alamarBlue assay. (c) Cationic hydrogels toward HaCaT cells determined by the XTT assay and (d) hDF cells using the alamarBlue assay. All data are shown as a mean value ± SD (*n* = 6).

To confirm that the wound dressing materials did not cause cytotoxic effects, two types of human skin cells, human keratinocytes (HaCaT) and human dermal fibroblasts (hDF), were used to evaluate the *in vitro* cytotoxicity of the amino-functional HBDLDs and the cationic dendritic hydrogels. The concentrations of the amino-functional HBDLDs were chosen based on the MIC values, and the cells and the amino-functional HBDLDs were incubated for 24 h at 37 °C. No significant cytotoxicity toward either HaCaT (Figure 5a) or hDF (Figure 5b) cells was found, as made evident by cell viability values above ∼80% (based on ISO 10993-5:2009)^46^ which indicates that the amino-functional HBDLDs are biocompatible at their MIC values. All cationic dendritic hydrogels exhibited good biocompatibility toward HaCaT (Figure 5c) and hDF (Figure 5d) cells, with cell viability above 80% after incubation with cells for 24 h. The G5 hydrogels exhibited better biocompatibility than G6 hydrogels at comparable PEG lengths, likely because the G6 hydrogels have greater cationic charges leading to stronger interactions with the cells. The H10K-G5 hydrogel formed from PEG10K-G5-NH_3_^+^ exhibited excellent biocompatibility toward both HaCaT (cell viability 101%) and hDF (cell viability 108%) cells. Potent and biocompatible, the H10K-G5 hydrogel was identified and used for the *in vitro* cell infection study and immunomodulation activities in HaCaT cells.

### Antibacterial Activity of the Amino-Functional Hydrogels

**Figure 6:**
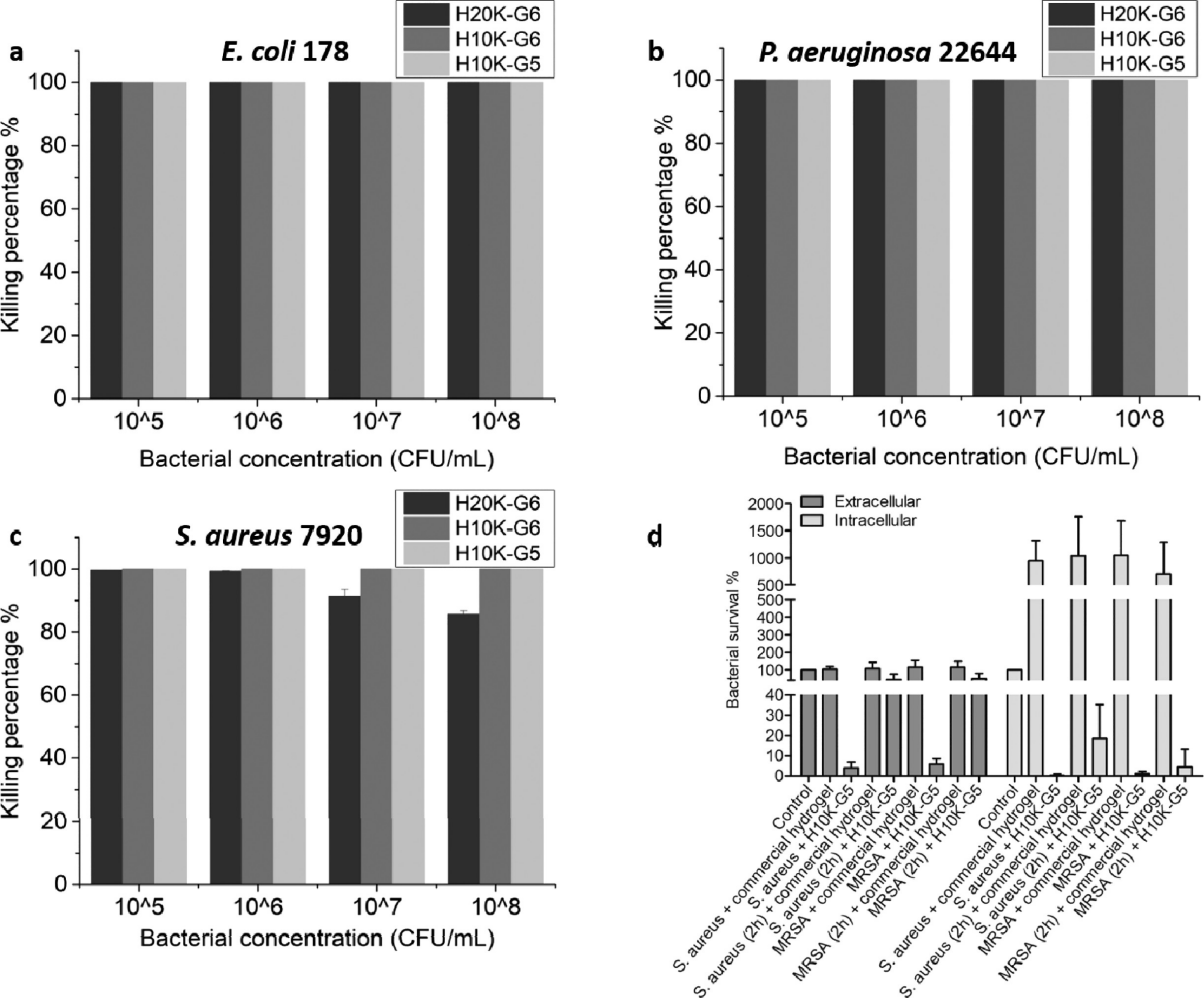
Antibacterial activity of hydrogels against (a) *E. coli* 178, (b) *P. aeruginosa* 22644, and (c) *S. aureus* 7920 after incubation with different bacterial concentrations for 6 h at 37 °C. (d) *In vitro* cell infection assay in HaCaT cells using H10K-G5 hydrogel and a commercial wound dressing hydrogel as control.

Hydrogels are excellent materials for wound dressing because of their soft and pliable tactile properties, which would provide ideal contact on the skin and maintain the moist environment beneficial for optimal wound healing. Hydrogels with inherent antibacterial properties are beneficial to wound healing^47^ because they can combat bacterial infections without the addition of extra antibacterial agents. The antibacterial properties of the cationic dendritic hydrogels toward *E. coli*, *P. aeruginosa*, and *S. aureus* were explored to assess this, where it was found that H10K-G6 and H10K-G5 exhibited 100% killing efficacy toward *E. coli* (Figure 6a), *S. aureus* (Figure 6c), and *P. aeruginosa* (Figure 6b) at all bacterial concentrations ranging from 10^5^ to 10^8^ CFU mL^–1^. The bacterial killing efficacy of H20K-G6 against *E. coli* (Figure 6a) and *P. aeruginosa* (Figure 6b) was also 100% over these bacterial concentrations. More than 99% of *S. aureus* was killed at concentrations of 10^5^ and 10^6^ CFU mL^–1^ (Figure 6c), and 93% and 87% of *S. aureus* were killed at concentrations of 10^7^ and 10^8^ CFU mL^–1^, respectively. These results suggest that the cationic dendritic hydrogels are effective as antibacterial materials for treating serious bacterial infections. As the hydrogels start to degrade within an hour, the effective antibacterial performance is probably the combination of dual actions from the hydrogels and their degradation products.

An *in vitro* cell infection study was performed by using HaCaT cells to assess the ability of the cationic dendritic hydrogels to treat infected skin cells. Keratinocytes, like HaCaT cells, are located at the outermost layer of the skin and constitute ∼90–95% of the epidermal cell population.^48^ Analyses of the intracellular and extracellular bacteria after treatment with H10K-G5 for 6 h was conducted and compared with a commercial hydrogel wound dressing. The extracellular and intracellular bacteria treated with H10K-G5 significantly decreased for both *S. aureus* and MRSA strains (Figure 6d). Bacterial survival percentages of above 100% were found for the commercial hydrogel wound dressing for both *S. aureus* and MRSA, suggesting that the commercial wound dressing is not able to kill or inhibit the growth of these bacteria (Figure 6d). Remarkably, intracellular and extracellular MRSA survival percentages were 1% and 6%, respectively, when HaCaT cells were infected at the same time that the cationic hydrogel was applied. When HaCaT cells were infected with bacteria 2 h before applying the cationic hydrogel, the intracellular and extracellular MRSA survival percentages were 9% and 51%, respectively. MRSA survival increased due to the proliferation of bacteria during the 2 h preinfection time, but the efficiency for treating the preinfected HaCaT is supposed to be improved by increasing the volume of the cationic hydrogel used to treat the bacteria. Other types of bacterial strains such as *E. coli*, *P. aeruginosa*, and GAS were also tested, and a significant decrease in the bacterial numbers after treatment with the H10K-G5 hydrogel (Figure S6) was found. These results serve to validate the ingenuity of these materials and their capacity to treat skin infections caused by various kinds of bacteria.

### Inhibition of *E. coli* Biofilm Formation

Inspired by the SEM images showing decreased *E. coli* fimbriae after treatment with amino-functional HBDLDs, we hypothesized that these cationic materials may be successful in affecting the formation of biofilms. To test this, the effects of the H10K-G5 hydrogel on biofilm formation using *E. coli* #12 and its isogenic strain lacking curli, a major biofilm forming fimbriae, were investigated.^49^ A significant reduction of new *E. coli* biofilm formation was found when they were treated with H10K-G5 using crystal violet assay (Figure S7). To further confirm the association of H10K-G5 with *E. coli* curli, an *E. coli* strain expressing curli, WE1*bcsA* (curli +/cellulose −), was used for the biofilm assay. The results show that treatment with H10K-G5 significantly reduces initial biofilm formation in curli positive strains (Figure S7). Although bacteria can attach on the cationic hydrogels due to the attraction of the opposite charges, the degradability of the hydrogels can efficiently prevent the formation of biofilm on the surface of the gels.

### Immune Modulation in HaCaT Cells

**Figure 7:**
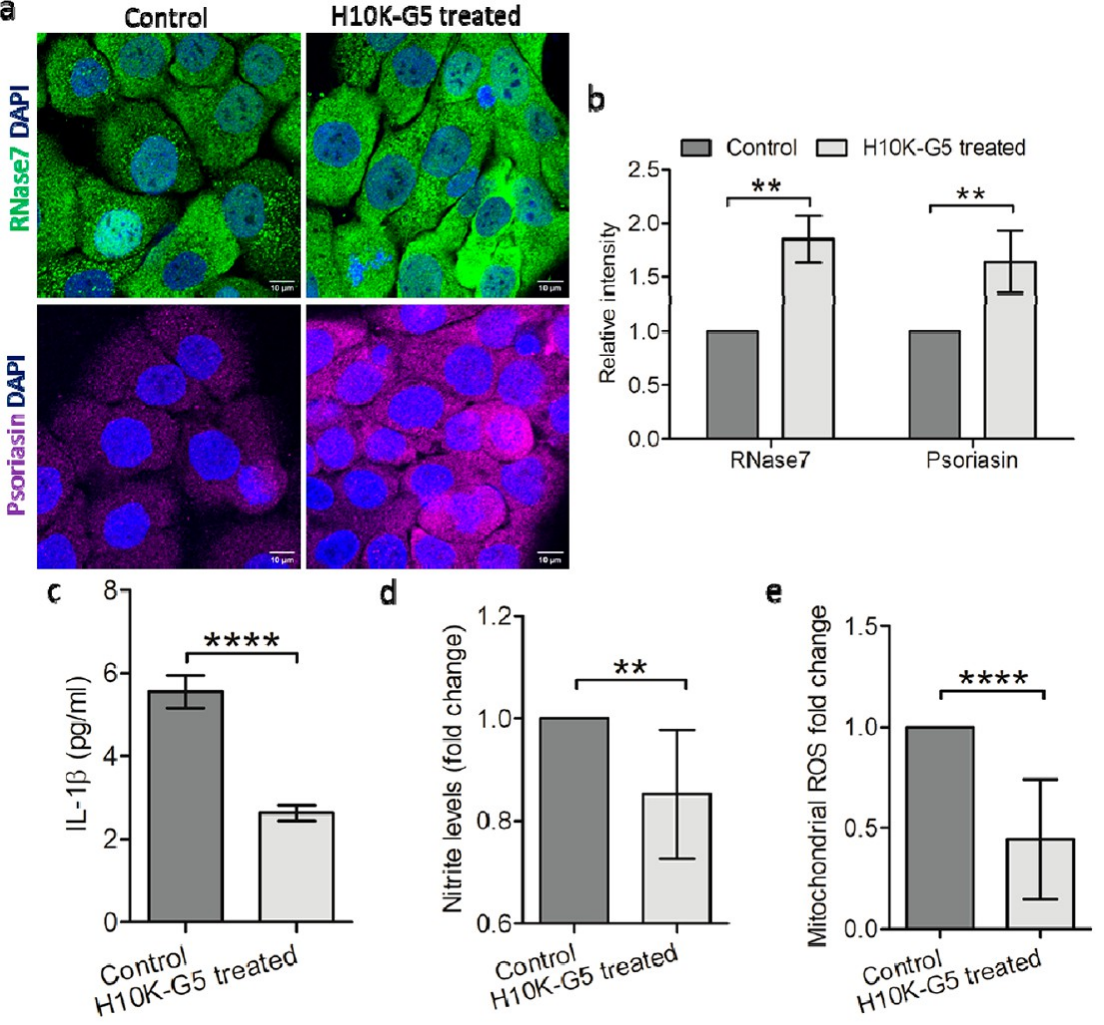
(a) Detection of the antimicrobial peptides, RNase7 and psoriasin using confocal microscopy, (b) relative densitometric analysis of RNase7 and psoriasin in HaCaT cells, (c) expression of proinflammatory cytokine IL-1β in HaCaT cells, (d) nitrite, and (e) mitochondrial ROS levels in *S. aureus*-infected HaCaT cells. All data are shown as mean values ± SD (*n* = 9 for b and c, *n* = 6 for d and e). Significance levels mentioned as **P < 0.01 and ****P < 0.0001.

When considering that the cationic hydrogels are primarily interacting with negatively charged bacterial surfaces, we hypothesized that bacteria within the cells might be killed by the antimicrobial peptides secreted by HaCaT cells themselves. The effects of cationic dendritic hydrogels on the immunomodulation activity of HaCaT cells were therefore investigated. Antimicrobial peptides RNase7 (gene: RNASE7) and psoriasin (gene: S100A7) are predominantly present in human skin. The expressions of RNASE7 and S100A7 were detected by using quantitative polymerase chain reaction (qPCR) with and without treatment of H10K-G5. The expressions of RNASE7 and S100A7 were constitutively expressed in the steady state and significantly upregulated when H10K-G5 was added (Figure S8a). Similarly, a significant increase in the expression of RNase7 and psoriasin occurred at the protein levels. Representative confocal microscopy images are shown in Figure 7a, and both the fluorescence of the RNase7 (Alexa 488, green fluorescence dye) and psoriasin (Alexa 647, bright, far-red fluorescent dye) within the cells were clearly enhanced after contact with H10K-G5 as verified by densitometric analysis (Figure 7b). The results suggest that cationic dendritic hydrogels not only kill bacteria through direct interaction but also trigger HaCaT cells to secret antimicrobial peptides to efficiently kill bacteria intracellularly. This alteration in gene expression could be due to a probable dual effect of cationic hydrogels and its degradation products.

In addition, the proinflammatory cytokine IL-1β and free radical species were also analyzed in *S. aureus* infected HaCaT cells (infected for 4 h). IL-1β was downregulated at both mRNA levels (Figure S8b) and the secreted protein (Figure 7c) after interaction with H10K-G5, as confirmed with ELISA from the supernatants. Free radical formation by the host cell is a host mechanism used to combat bacterial infections; however, an excess of free radicals also induces apoptotic cell death. A regulation of the balance between free radical species is therefore required for physiological activity of the cells. To verify the effect of H10K-G5 on free radical species in HaCaT cells after infection with *S. aureus* for 4 h, both reactive nitrogen species (NO) and reactive oxygen species (ROS) were analyzed. The infected HaCaT cells treated with H10K-G5 showed reduced NO (Figure 7d) and mitochondrial ROS (Figure 7e) levels in comparison to the untreated infected cells as the control, and relative densitometry analyses of mitochondrial ROS showed lower fluorescence levels upon hydrogel treatment (Figure S8c,d), suggesting minimal imposed adverse effects as increased mitochondrial ROS in keratinocytes might cause cell death via the induction of apoptosis.^50,51^ The decreased NO and mitochondrial ROS levels also correspond with less severe bacterial infections for HaCaT cells treated with H10K-G5. In addition, the total ROS level (Figure S9) increased after treatment with H10K-G5, likely due to the increased expression of antimicrobial peptide psoriasin in keratinocytes.^52,53^ The decreased IL-1β indicates that the cationic dendritic hydrogels could likely prevent inflammatory responses, while the decreased free radical species suggests that the cationic materials could probably prevent oxidative stress in *S. aureus*-infected HaCaT cells.

## Conclusion

In summary, a new class of cationic dendritic hydrogels with inherent antibacterial properties were developed and tested against both Gram-positive and Gram-negative clinical strains. The hydrogels performed outstandingly at eliminating bacterial infections caused by clinical drug-resistant bacteria and were able to inhibit *E. coli* biofilm formation. With their excellent biocompatibility, degradability, anti-inflammatory properties, and the ability to induce host-mediated bacterial killing by enhancing the expression of the antimicrobial peptides in HaCaT cells, these cationic dendritic hydrogels present themselves as promising wound dressing materials to treat skin infections caused by wound-isolated MDR bacteria.

## Supporting Information Available

Detailed experimental procedures, synthesis and characterization of materials, antibacterial and antibiofilm results of the materials, expression of RNASE7, S100A7, IL1B mRNA, and ROS levels, Figures S1–S9 (PDF)
Video S1 (MP4)
