# Supplementary material for: Dendritic Hydrogels Induce Immune Modulation in Human Keratinocytes and Effectively Eradicate Bacterial Pathogens
Source: J Am Chem Soc. 2021 Oct 12;143(41):17180–90. doi: 10.1021/jacs.1c07492 (PMC8532153; doi:10.1021/jacs.1c07492)
Supplement: Supplementary file 1 — ja1c07492_si_002.pdf [file ja1c07492_si_002.pdf]

SUPPLEMENTARY MATERIALS

Dendritic hydrogels induce immune modulation in human keratinocytes and effectively eradicate bacterial pathogens

Yanmiao Fan[a,§], Soumitra Mohanty[b,c,§], Yuning Zhang[a], Mads Lüchow[a], Liguo Qin[a,d], Lisa Fortuin[a], Annelie Brauner[b,c]* and Michael Malkoch[a]*

[a] KTH Royal Institute of Technology, School of Chemical Science and Engineering, Fiber and Polymer Technology, Teknikringen 56-58, SE-100 44 Stockholm, Sweden

[b] Department of Microbiology, Tumor and Cell Biology, Karolinska Institutet, SE-17165 Stockholm, Sweden

[c] Division of Clinical Microbiology, Karolinska University Hospital, Solna, SE-17176 Stockholm, Sweden

[d] Institute of Design Science and Basic Components, School of Mechanical Engineering, Xi´an Jiaotong University, 710049 Xi´an, PR China

[§] These authors contributed equally and shared first authorship

* These authors contributed equally and are shared last and corresponding authors. Email addresses: malkoch@kth.se; annelie.brauner@ki.se

Supplementary Methods

Materials and Methods

Materials

All materials and solvents were purchased from commercial sources and were used as received unless otherwise stated. Hydroxy-functional polyethylene glycol (PEG) PEG6K-OH, PEG10K-OH, PEG20K-OH, phosphate buffered saline (PBS) tablets (pH 7.4, the total ion concentration was approximately 0.15 M), cesium fluoride, β-alanine and trifluoroacetic acid (TFA) were purchased from Sigma-Aldrich. 2,2-Bis(hydroxymethyl)propionic acid (bis-MPA) was obtained from Perstorp AB, Sweden. Boc-β-alanine was obtained from Alfa Aesar. 1,1'-Carbonyldiimidazole (CDI) was obtained from Carbosynth. Deuterated solvents were from Cambridge Isotope Laboratories. *p*-Toluenesulfonic acid (*p*TSA, 98%) and dichloromethane (DCM) were purchased from Merck. Mueller-Hinton broth (MHB II) was from Fluka. The commercial Microdacyn Hydrogel containing hypochlorous acid and sodium hypochlorite as antibacterial agents was bought from Sonoma Pharmaceuticals.

Bacterial strains

Escherichia coli 208 (*E. coli* 208) and *Escherichia coli* 178 (*E. coli* 178) were kindly provided by Professor Paul Orndorff from North Carolina State University. *Staphylococcus aureus* 2569 (*S. aureus* 2569) and *Pseudomonas aeruginosa* 22644 (*P. aeruginosa* 22644) were obtained from DSMZ. *Escherichia coli #12* (*E. coli #12*), *Escherichia coli* AB1 (*E. coli* AB1), methicillin resistant *Staphylococcus aureus* (MRSA) CCUG 31966, Group A streptococcus (GAS) ATCC 19615, *Pseudomonas aeruginosa* AB2 (*P. aeruginosa* AB2) and *Staphylococcus aureus* 7920 (*S. aureus* 7920) were clinical isolate strains from Karolinska University Hospital.

Synthesis of hydroxy-functional HBDLDs[1]

Synthesis of PEG6K-G5-OH. Prepared according to the general polycondensation reaction between bis-MPA and PEG, with reactants PEG6K-OH (1.33 mmol, 8.00 g), bis-MPA (82.7 mmol, 11.1 g) and *p*TSA (5 wt.%, 554 mg). The product was collected as white solid. ^{1}H NMR (400 MHz, Methanol-d$_4$): δ (ppm) 4.40−4.17 (q, 124H, -CH_2-OCO-, bis-MPA), 3.66 (q, 557H, CH_2-CH_2-O-, PEG), 1.40−1.14 (q, 186H, -CH_3, bis-MPA). ^{13}C NMR (100 MHz, DMSO-D$_6$): δ (ppm) 174.2−171.6 (1C, -COO-, bis-MPA), 69.75 (2C, CH_2-CH_2-O-, PEG), 65.4−63.6 (1C, -CH_2-, bis-MPA), 50.2 (1C, -COO-C-((CH_2-OH)$_2$, CH_3), bis-MPA), 49.5 (1C, -COOH-C-((CH_2-OR)$_2$, CH_3), bis-MPA), 48.1 (1C, -COO-C-(CH_2-OH, CH_3, CH_2-OR), bis-MPA), 46.2-45.5 (1C, -COO-C-((CH_2-OR)$_2$, CH_3), bis-MPA), 16.8-16.7 (1C, -CH_3, bis-MPA).

Synthesis of PEG6K-G6-OH. Prepared according to the general polycondensation reaction between bis-MPA and PEG, with reactants PEG6K-OH (1.00 mmol, 6.00 g), bis-MPA (126 mmol, 16.9 g) and *p*TSA (5 wt.%, 845 mg). The product was collected as milky solid (83%, 6.33 g). ^{1}H NMR (400 MHz, Methanol-d$_4$): δ (ppm) 4.40-4.2 (q, 252H, -CH_2-OCO-, bis-MPA), 3.66 (q, 557H, CH_2-CH_2-O-, PEG), 1.38-1.14 (q, 378H, -CH_3, bis-MPA). ^{13}C NMR (100 MHz, DMSO-D$_6$): δ (ppm) 174.2−171.6 (1C, -COO-, bis-MPA), 69.75 (2C, CH_2-CH_2-O-, PEG), 65.4−63.6 (1C, -CH_2-, bis-MPA), 50.2 (1C, -COO-C-((CH_2-OH)$_2$, CH_3), bis-MPA), 49.5 (1C, -COOH-C-((CH_2-OR)$_2$, CH_3), bis-MPA), 48.1 (1C, -COO-C-(CH_2-OH, CH_3, CH_2-OR), bis-MPA), 46.2-45.5 (1C, -COO-C-((CH_2-OR)$_2$, CH_3), bis-MPA), 16.8-16.7 (1C, -CH_3, bis-MPA).

Synthesis of PEG10K-G5-OH. Prepared according to the general polycondensation reaction between bis-MPA and PEG, with reactants PEG10K-OH (1.00 mmol, 10.00 g), bis-MPA (62 mmol, 8.31 g) and *p*TSA (5 wt.%, 415.5 mg). The product was collected as fluffy solid. ^{1}H NMR (400 MHz, Methanol-d$_4$): δ (ppm) 4.40-4.18 (q, 124H, -CH_2-OCO-, bis-MPA), 3.66 (q, 909H, CH_2-CH_2-O-, PEG), 1.38−1.13 (q, 186H, -CH_3, bis-MPA). ^{13}C NMR (100 MHz, DMSO-D$_6$): δ (ppm) 174.2−171.6 (1C, -COO-, bis-MPA), 69.75 (2C, CH_2-CH_2-O-, PEG), 65.4−63.6 (1C, -CH_2-, bis-MPA), 50.2 (1C, -COO-C-((CH_2-OH)$_2$, CH_3), bis-MPA), 49.5 (1C, -COOH-C-((CH_2-OR)$_2$, CH_3),

bis-MPA), 48.1 (1C, -COO-C-(CH_2-OH, CH_3, CH_2-OR), bis-MPA), 46.2-45.5 (1C, -COO-C-((CH_2-OR)$_2$, CH_3), bis-MPA), 16.8-16.7 (1C, -CH_3, bis-MPA).

Synthesis of PEG10K-G6-OH. Prepared according to the general polycondensation reaction between bis-MPA and PEG, with reactants PEG10K-OH (1.00 mmol, 10.00 g), bis-MPA (125.2 mmol, 16.88 g) and *p*TSA (5 wt.%, 844 mg). The product was collected as white fluffy, a bit sticky solid (83%, 6.33 g). ^{1}H NMR (400 MHz, Methanol-d$_4$): δ (ppm) 4.40-4.18 (q, 252H, -CH_2-OCO-, bis-MPA), 3.66 (q, 909H, CH_2-CH_2-O-, PEG), 1.39-1.10 (q, 378H, -CH_3, bis-MPA). ^{13}C NMR (100 MHz, DMSO-D$_6$): δ (ppm) 174.2−171.6 (1C, -COO-, bis-MPA), 69.75 (2C, CH_2-CH_2-O-, PEG), 65.4−63.6 (1C, -CH_2-, bis-MPA), 50.2 (1C, -COO-C-((CH_2-OH)$_2$, CH_3), bis-MPA), 49.5 (1C, -COOH-C-((CH_2-OR)$_2$, CH_3), bis-MPA), 48.1 (1C, -COO-C-(CH_2-OH, CH_3, CH_2-OR), bis-MPA), 46.2-45.5 (1C, -COO-C-((CH_2-OR)$_2$, CH_3), bis-MPA), 16.8-16.7 (1C, - CH_3, bis-MPA).

Synthesis of PEG20K-G5-OH. Prepared according to the general polycondensation reaction between bis-MPA and PEG, with reactants PEG20K-OH (0.50 mmol, 10.00 g), bis-MPA (31.0 mmol, 4.16 g) and *p*TSA (5 wt.%, 208 mg). The product was collected as white solid. ^{1}H NMR (400 MHz, Methanol-d$_4$): δ (ppm) 4.40−4.2 (q, 124H, -CH_2-OCO-, bis-MPA), 3.66 (q, 1828H, CH_2-CH_2-O-, PEG), 1.38−1.13 (q, 186H, -CH_3, bis-MPA). ^{13}C NMR (100 MHz, DMSO-D$_6$): δ (ppm) 173.8−172.6 (1C, -COO-, bis-MPA), 69.71 (2C, CH_2-CH_2-O-, PEG), 65.4−63.6 (1C, -CH_2-, bis-MPA), 50.2 (1C, -COO-C-((CH_2-OH)$_2$, CH_3), bis-MPA), 49.5 (1C, -COOH-C-((CH_2-OR)$_2$, CH_3), bis-MPA), 48.1 (1C, -COO-C-(CH_2-OH, CH_3, CH_2-OR), bis-MPA), 46.2-45.5 (1C, -COO-C-((CH_2-OR)$_2$, CH_3), bis-MPA), 16.8-16.7 (1C, -CH_3, bis-MPA).

Synthesis of PEG20K-G6-OH. Prepared according to the general polycondensation reaction between bis-MPA and PEG, with reactants PEG20K-OH (0.50 mmol, 10.00 g), bis-MPA (63.0 mmol, 8.45 g) and *p*TSA (5 wt.%, 422 mg). The product was collected as white solid. ^{1}H NMR (400 MHz, Methanol-d$_4$): δ (ppm) 4.45−4.18 (q, 252H, -CH_2-OCO-, bis-MPA), 3.66 (q, 1828H, CH_2-CH_2-O-, PEG), 1.4-1.18 (q, 378H, -CH_3, bis-MPA). ^{13}C NMR (100 MHz, DMSO-D$_6$):

δ (ppm) 174.2−171.6 (1C, -COO-, bis-MPA), 69.75 (2C, CH_2-CH_2-O-, PEG), 65.4−63.6 (1C, -CH_2-, bis-MPA), 50.2 (1C, -COO-C-((CH_2-OH)$_2$, CH_3), bis-MPA), 49.5 (1C, -COOH-C-((CH_2-OR)$_2$, CH_3), bis-MPA), 48.1 (1C, -COO-C-(CH_2-OH, CH_3, CH_2-OR), bis-MPA), 46.2-45.5 (1C, -COO-C-((CH_2-OR)$_2$, CH_3), bis-MPA), 16.8-16.7 (1C, -CH_3, bis-MPA).

Synthesis of boc-protected amino-functional HBDLDs

Boc-protected β-alanine was dissolved in DCM (final concentration of 1 M), and was then activated using CDI. The reaction was left to proceed for 1 h to allow the formation of the imidazolide-activated intermediate, after which hydroxy-terminated HBDLDs and cesium fluoride were added into the vessel. The reaction was then left to react for 14 h and the mixture was quenchi with water. The reaction mixture was diluted with DCM and washed 3 times with 10% aqueous $NaHSO_4$, 3 times with 10% aqueous $NaHCO_3$ and once with brine. And the solvent was removed using evaporator and vacuum.

Synthesis of PEG6K-G5-NH-BOC. Boc-protected β-alanine (20 mmol, 3784.2 mg) was dissolved in 44 mL DCM in a round-bottom flask equipped with magnetic stir bar. CDI (20 mmol, 3243 mg) was then added and the reaction was left to proceed for 1 h. Then, PEG6K-G5-OH (0.156 mmol, 2063.27 mg) and cesium fluoride (2 mmol, 303.8 mg) were added and the reaction was allowed to proceed overnight (14 h). ^{13}C NMR (101 MHz, $CDCl_3$): δ (ppm) 171.80 (-**C**OO-, bis-MPA), 155.81 (-**C**OO-, β-alanine), 79.27 (-COO-**C**- (CH_3)$_3$, BOC), 70.54 (CH_2-CH_2-O-, PEG), 64.89 (-**C**H_2-, bis-MPA), 46.47 (-COO-**C**-(CH_2)$_2$, bis-MPA), 36.09 (-COO-**C**H_2-CH_2-NH-, β-alanine), 34.39 (-COO-CH_2-**C**H_2-NH-, β-alanine), 28.37 (-CH_3, BOC), 17.64 (-CH_3, bis-MPA).

Synthesis of PEG6K-G6-NH-BOC. Boc-protected β-alanine (20 mmol, 3784.2 mg) was dissolved in 44 mL DCM in a round-bottom flask equipped with magnetic stir bar. CDI (20 mmol, 3243 mg) was then added and the reaction was left to proceed for 1h. Then, PEG6K-G6-OH (0.078 mmol, 1612.3 mg) and cesium fluoride (2 mmol, 303.8 mg) were added and the reaction was allowed to proceed overnight (14 h) with stirring. ^{13}C NMR (101 MHz, $CDCl_3$): δ (ppm) 171.82 (-**C**OO-, bis-

MPA), 155.85 (-**C**OO-, β-alanine), 79.30 (-COO-**C**- $(CH_3)_3$, BOC), 70.55 (CH_2-CH_2-O-, PEG), 64.92 (-**C**H_2-, bis-MPA), 46.51 (-COO-**C**-$(CH_2)_2$, bis-MPA), 36.12 (-COO-**C**H_2-CH_2-NH-, β-alanine), 34.42 (-COO-CH_2-**C**H_2-NH-, β-alanine), 28.39 (-CH_3, BOC), 17.66 (-CH_3, bis-MPA).

Synthesis of PEG10K-G5-NH-BOC. Boc-protected β-alanine (20 mmol, 3784.2 mg) was dissolved in 44 mL DCM in a round-bottom flask equipped with magnetic stir bar. CDI (20 mmol, 3243 mg) was then added and the reaction was left to proceed for 1h. Then, PEG10K-G5-OH (0.156 mmol, 2688.3 mg) and cesium fluoride (2 mmol, 303.8 mg) were added and the reaction was allowed to proceed overnight (14 h) with stirring. ^{13}C NMR (101 MHz, $CDCl_3$): δ (ppm) 171.75 (-**C**OO-, bis-MPA), 155.79 (-**C**OO-, β-alanine), 79.24 (-COO-**C**- $(CH_3)_3$, BOC), 70.52 (CH_2-CH_2-O-, PEG), 64.88 (-**C**H_2-, bis-MPA), 46.45 (-COO-**C**-$(CH_2)_2$, bis-MPA), 36.08 (-COO-**C**H_2-CH_2-NH-, β-alanine), 34.38 (-COO-CH_2-**C**H_2-NH-, β-alanine), 28.36 (-CH_3, BOC), 17.63 (-CH_3, bis-MPA).

Synthesis of PEG10K-G6-NH-BOC. Boc-protected β-alanine (20 mmol, 3784.2 mg) was dissolved in 44 mL DCM in a round-bottom flask equipped with magnetic stir bar. CDI (20 mmol, 3243 mg) was then added and the reaction was left to proceed for 1h. Then, PEG10K-G6-OH (0.078 mmol, 1924.7 mg) and cesium fluoride (2 mmol, 303.8 mg) were added and the reaction was allowed to proceed overnight (14 h) with stirring. ^{13}C NMR (101 MHz, $CDCl_3$): δ (ppm) 171.78 (-**C**OO-, bis-MPA), 155.83 (-**C**OO-, β-alanine), 79.27 (-COO-**C**- $(CH_3)_3$, BOC), 70.56 (CH_2-CH_2-O-, PEG), 64.90 (-**C**H_2-, bis-MPA), 46.47 (-COO-**C**-$(CH_2)_2$, bis-MPA), 36.10 (-COO-**C**H_2-CH_2-NH-, β-alanine), 34.41 (-COO-CH_2-**C**H_2-NH-, β-alanine), 28.39 (-CH_3, BOC), 17.67 (-CH_3, bis-MPA).

Synthesis of PEG20K-G5-NH-BOC. Boc-protected β-alanine (20 mmol, 3784.2 mg) was dissolved in 44 mL DCM in a round-bottom flask equipped with magnetic stir bar. CDI (20 mmol, 3243 mg) was then added and the reaction was left to proceed for 1h. Then, PEG20K-G5-OH (0.156 mmol, 4250.77 mg) and cesium fluoride (2 mmol, 303.8 mg) were added and the reaction was allowed to proceed overnight (14 h) with stirring. ^{13}C NMR (101 MHz, $CDCl_3$): δ (ppm) 171.78 (-**C**OO-, bis-MPA), 155.84 (-**C**OO-, β-alanine), 79.30 (-COO-**C**- $(CH_3)_3$, BOC), 70.53

(CH_2-CH_2-O-, PEG), 64.92 (-**C**H_2-, bis-MPA), 46.50 (-COO-**C**-(CH_2)$_2$, bis-MPA), 36.09 (-COO-**C**H_2-CH_2-NH-, β-alanine), 34.39 (-COO-CH_2-**C**H_2-NH-, β-alanine), 28.38 (-CH_3, BOC), 17.65 (-CH_3, bis-MPA).

Synthesis of PEG20K-G6-NH-BOC. Boc-protected β-alanine (20 mmol, 3784.2 mg) was dissolved in 44 mL DCM in a round-bottom flask equipped with magnetic stir bar. CDI (20 mmol, 3243 mg) was then added and the reaction was left to proceed for 1h. Then, PEG20K-G6-OH (0.078 mmol, 2706.04 mg) and cesium fluoride (2 mmol, 303.8 mg) were added and the reaction was allowed to proceed overnight (14 h) with stirring. ^{13}C NMR (101 MHz, $CDCl_3$): δ (ppm) 171.77 (-**C**OO-, bis-MPA), 155.84 (-**C**OO-, β-alanine), 79.29 (-COO-**C**- (CH_3)$_3$, BOC), 70.55 (CH_2-CH_2-O-, PEG), 64.91 (-**C**H_2-, bis-MPA), 46.51 (-COO-**C**-(CH_2)$_2$, bis-MPA), 36.09 (-COO-**C**H_2-CH_2-NH-, β-alanine), 34.40 (-COO-CH_2-**C**H_2-NH-, β-alanine), 28.38 (-CH_3, BOC), 17.63 (-CH_3, bis-MPA).

Deprotection of the boc-protected amino-functional HBDLDs

Boc-protected amino-functional HBDLDs were dissolved in trifluoroacetic acid and the reaction was allowed to proceed for 1.5 h. The solvent was removed by rotor evaporator, and the mixture was dissolved in methanol and precipitated in cold ether twice. The remaining trace solvents were removed by vacuum.

Synthesis of PEG6K-G5-NH_3^+. ^1H NMR (400 MHz, Methanol-d_4): δ (ppm) 8.04 (NH_3^+, β-alanine), 4.19 (-CH_2-, bis-MPA), 3.51 (CH_2-CH_2-O-, PEG), 3.04 (-COO-CH_2-C**H**$_2$-NH-, β-alanine), 2.68 (-COO-C**H**$_2$-CH_2-NH-, β-alanine), 1.18 (-CH_3, bis-MPA). ^{13}C NMR (101 MHz, MeOD): δ (ppm) 171.98 (-**C**OO-, bis-MPA), 170.44 (-**C**OO-, β-alanine), 69.89 (CH_2-CH_2-O-, PEG), 65.49 (-**C**H_2-, bis-MPA), 46.49 (-COO-**C**-(CH_2)$_2$, bis-MPA), 34.98 (-COO-**C**H_2-CH_2-NH-, β-alanine), 30.82 (-COO-CH_2-**C**H_2-NH-, β-alanine), 16.70 (-CH_3, bis-MPA).

Synthesis of PEG6K-G6-NH_3^+.^1H NMR (400 MHz, Methanol-d_4): δ (ppm) 8.04 (NH_3^+, β-alanine), 4.19 (-CH_2-, bis-MPA), 3.51 (CH_2-CH_2-O-, PEG), 3.04 (-COO-CH_2-C**H**$_2$-NH-, β-alanine), 2.68 (-

COO-C**H**$_2$-CH$_2$-NH-, β-alanine), 1.18 (-CH$_3$, bis-MPA). ^{13}C NMR (101 MHz, MeOD): δ (ppm) 172.06 (-**C**OO-, bis-MPA), 170.43 (-**C**OO-, β-alanine), 69.90 (CH$_2$-CH$_2$-O-, PEG), 65.49 (-**C**H$_2$-, bis-MPA), 46.47 (-COO-**C**-(CH$_2$)$_2$, bis-MPA), 34.94 (-COO-**C**H$_2$-CH$_2$-NH-, β-alanine), 30.80 (-COO-CH$_2$-**C**H$_2$-NH-, β-alanine), 16.70 (-CH$_3$, bis-MPA).

Synthesis of PEG10K-G5-NH$_3^+$. ^{1}H NMR (400 MHz, Methanol-d$_4$): δ (ppm) 8.04 (NH$_3^+$, β-alanine), 4.19 (-CH$_2$-, bis-MPA), 3.51 (CH$_2$-CH$_2$-O-, PEG), 3.04 (-COO-CH$_2$-C**H**$_2$-NH-, β-alanine), 2.68 (-COO-C**H**$_2$-CH$_2$-NH-, β-alanine), 1.18 (-CH$_3$, bis-MPA). ^{13}C NMR (101 MHz, MeOD): δ (ppm) 171.97 (-**C**OO-, bis-MPA), 170.45 (-**C**OO-, β-alanine), 69.89 (CH$_2$-CH$_2$-O-, PEG), 65.94 (-**C**H$_2$-, bis-MPA), 46.50 (-COO-**C**-(CH$_2$)$_2$, bis-MPA), 35.02 (-COO-**C**H$_2$-CH$_2$-NH-, β-alanine), 30.83 (-COO-CH$_2$-**C**H$_2$-NH-, β-alanine), 16.73 (-CH$_3$, bis-MPA).

Synthesis of PEG10K-G6-NH$_3^+$. ^{1}H NMR (400 MHz, Methanol-d$_4$): δ (ppm) 8.04 (NH$_3^+$, β-alanine), 4.19 (-CH$_2$-, bis-MPA), 3.51 (CH$_2$-CH$_2$-O-, PEG), 3.04 (-COO-CH$_2$-C**H**$_2$-NH-, β-alanine), 2.68 (-COO-C**H**$_2$-CH$_2$-NH-, β-alanine), 1.18 (-CH$_3$, bis-MPA). ^{13}C NMR (101 MHz, MeOD): δ (ppm) 171.99 (-**C**OO-, bis-MPA), 170.44 (-**C**OO-, β-alanine), 69.90 (CH$_2$-CH$_2$-O-, PEG), 65.49 (-**C**H$_2$-, bis-MPA), 46.49 (-COO-**C**-(CH$_2$)$_2$, bis-MPA), 34.96 (-COO-**C**H$_2$-CH$_2$-NH-, β-alanine), 30.80 (-COO-CH$_2$-**C**H$_2$-NH-, β-alanine), 16.70 (-CH$_3$, bis-MPA).

Synthesis of PEG20K-G5-NH$_3^+$. ^{1}H NMR (400 MHz, Methanol-d$_4$): δ (ppm) 7.97 (NH$_3^+$, β-alanine), 4.19 (-CH$_2$-, bis-MPA), 3.51 (CH$_2$-CH$_2$-O-, PEG), 3.04 (-COO-CH$_2$-C**H**$_2$-NH-, β-alanine), 2.68 (-COO-C**H**$_2$-CH$_2$-NH-, β-alanine), 1.18 (-CH$_3$, bis-MPA). ^{13}C NMR (101 MHz, MeOD): δ (ppm) 171.93 (-**C**OO-, bis-MPA), 170.44 (-**C**OO-, β-alanine), 69.96 (CH$_2$-CH$_2$-O-, PEG), 65.46 (-**C**H$_2$-, bis-MPA), 46.53 (-COO-**C**-(CH$_2$)$_2$, bis-MPA), 35.09 (-COO-**C**H$_2$-CH$_2$-NH-, β-alanine), 30.82 (-COO-CH$_2$-**C**H$_2$-NH-, β-alanine), 16.77 (-CH$_3$, bis-MPA).

Synthesis of PEG20K-G6-NH$_3^+$. ^{1}H NMR (400 MHz, Methanol-d$_4$): δ (ppm) 8.01 (NH$_3^+$, β-alanine), 4.19 (-CH$_2$-, bis-MPA), 3.51 (CH$_2$-CH$_2$-O-, PEG), 3.04 (-COO-CH$_2$-C**H**$_2$-NH-, β-alanine), 2.68 (-COO-C**H**$_2$-CH$_2$-NH-, β-alanine), 1.18 (-CH$_3$, bis-MPA). ^{13}C NMR (101 MHz, MeOD): δ

(ppm) 171.97 (-**C**OO-, bis-MPA), 170.49 (-**C**OO-, β-alanine), 69.98 (CH_2-CH_2-O-, PEG), 65.50 (-**C**H_2-, bis-MPA), 46.52 (-COO-**C**-$(CH_2)_2$, bis-MPA), 35.10 (-COO-**C**H_2-CH_2-NH-, β-alanine), 30.84 (-COO-CH_2-**C**H_2-NH-, β-alanine), 16.77 (-CH_3, bis-MPA).

Synthesis of PEG10K-COO-NHS. PEG10K-COOH (10 g, 0.98 mmol) was dissolved in 4 mL DCM in a round-bottom flask. 1,1'-Carbonyldiimidazole (3.2 g, 19.6 mmol) was slowly added under N_2 flow and stirring and was allowed to react for 1 h at room temperature. *N*-Hydroxysuccinimide ester (2.7 g, 23.5 mmol) was added and the reaction was left to proceed for 14 h. The crude reaction mixture was diluted with DCM to a total volume of 50 mL and washed with $NaHSO_4$ twice. The crude product was dried using $MgSO_4$, filtered and concentrated to a total volume of 5 mL, and was then precipitated in ether. After removing the solvent, the product was collected as white powder. ^{1}H NMR (400 MHz, $CDCl_3$) δ 4.27 (t, J = 4.7 Hz, 6H), 3.64 (s, 1480H), 2.96 (t, J = 7.0 Hz, 4H), 2.83 (s, 8H), 2.77 (t, J = 7.0 Hz, 3H). ^{13}C NMR (101 MHz, $CDCl_3$) δ 170.79, 168.85, 167.55, 70.48, 68.82, 64.07, 28.55, 26.17, 25.45.

Formation of amine-functional hydrogels

Amino-functional HBDLDs were dissolved in deionized water, and 1M $NaHCO_3$ was added to neutralize the pH of the solution. The crosslinker, PEG10K-NHS, was dissolved in deionized water in a separate vial. These solutions were then transferred into one vial and mixed fully using a pipette. Most hydrogel gelation occurred within a few seconds. 8 amines out of the total 64 (G5) and 128 (G6) amines were used for crosslinking with a *N*-hydroxysuccinimide (NHS) functional PEG (PEG10K-NHS) crosslinker, and all hydrogels had solid contents of 10 wt.%.

Rheology

Rheological measurements were perfomed on a TA Discovery Hybrid 2 (DHR-2) rheometer equipped with a Peltier plate setup for temperature control. Crosslinking and degradation were analysed using an Ø 20 mm stainless steel Peltier plate-plate configuration, with time sweeps using strain (ϒ) = 1% and frequency (ω) = 1 Hz. All hydrogels were formed on the stage with premixing

for 2 s before the time sweep was started. Crosslinking time sweeps were conducted at 25 °C. Degradation experiments were conducted in phosphate buffered saline (PBS) pH 7.4 (the total ion concentration of PBS solution was approximately 0.15 M) at 37°C with a dynamic axial force control set to 0.2 N.

Scanning Electron Microscopy (SEM).

To observe the porous structure of the cationic hydrogels, the hydrogels were characterized using SEM after gelation. The hydrogels were freeze-dried and fractured with a sharp razor, then coated with 10 nm of Pt in a 208HR high-resolution sputter coater (Cressington, Watford, UK). SEM analysis was conducted using S-4800 field emission scanning electron microscope (Hitachi, Tokyo, Japan). For the preparation of the bacterial samples, bacteria in log phase was diluted with Tryptic Soy Broth (TSB) at 10^8 CFU mL^{-1}. A filter paper was placed in 1 mL bacterial solution and incubated at 37 °C for 24 h with slow shaking. The filter paper was washed gently to remove unattached bacteria and transferred to a vial containing 4 mL 250 µg mL^{-1} PEG10K-G5-NH_3^+, PBS buffer (pH 7.4) treated bacteria was used as a control. The vials were incubated at 37 °C for 6 h, after which the samples were washed in PBS and dipped into 2.5% glutaraldehyde in PBS overnight at 4 °C to fix the bacteria. This was subsequently followed by rinsing in PBS, and dehydration in 30%, 50%, 70%, 80%, 90% ethanol for 15 min. Finally, the samples were placed in absolute ethanol for 15 min (twice in absolute ethanol). The samples were dried using a critical point dryer (CPD) before being coated with 10 nm of Pt in a 208HR high-resolution sputter coater. Analysis was conducted using S-4800 field emission scanning electron microscope (Hitachi, Tokyo, Japan).

Nuclear magnetic resonance (NMR)

Analyses were performed using a Bruker AM NMR. ^1H NMR, ^{13}C NMR and ^{19}F NMR were recorded at 400 MHz, 101 MHz and 376 MHz, respectively. ^1H NMR spectra were acquired using a spectral window of 20 ppm, a relaxation delay of 1 s and 128 scans with automatic lock and shimming at a concentration of 10 mg mL^{-1}. ^{13}C NMR spectra were acquired using a spectral

window of 240 ppm, a relaxation delay of 2 s and 6000 scans at a concentration of 100 mg mL^{-1}. ^{19}F NMR were acquired using a spectral window of 237 ppm, a relaxation delay of 1 s and 500 scans at a concentration of 30 mg mL^{-1}. Analyses of the obtained spectra were conducted using MestreNova version 9.0 (Mestrelab Research S.L 2014).

Human cell lines and culture conditions

Human dermal fibroblast (hDF) were purchased from the American Tissue Culture Collection (ATCC). hDF cells were maintained in tissue culture flasks at 37 °C in CO_2 (5 %) with Dulbecco's Modified Eagle Medium (DMEM) supplemented with Fetal Bovine Serum 10% (v/v), L-glutamine (4 mM), penicillin (100 IU mL^{-1}) and streptomycin (100 µg mL^{-1}). HaCaT cells (Kind gift from Professor Mona Ståhle) were cultured in DMEM (Life Technologies) supplemented with 10% fetal bovine serum (Life Technologies) at 37 °C and 5% CO_2.

Cytotoxicity assay

Human dermal fibroblast (hDF) and human keratinocyte (HaCaT) cell lines were used for the cytotoxicity assay. Cells were harvested and transferred into 96-well plates (for copolymers) or 48-well plates (for hydrogels) at a concentration of 1×10^4 cells per well in 100 mL DMEM cultured for 24 h before use. The amino-functional HBDLDs were dissolved in the culture medium at the desired concentrations and were introduced to the cells and incubated for 24 h (37 °C, 5% CO_2). For the hydrogels, the cationic hydrogels (30 µL final volume) were first equilibrated with sterilized DI water for 20 min before being added to the 48-well plates (containing 600 µL culture medium), and incubated with the cells for 24 h (37 °C, 5% CO_2).

To determine cell viability cytotoxicity testing were performed with Alamar Blue or XTT assays. Alamar Blue was added and the incubation was continued for 4 h (37 °C, 5% CO_2). The fluorescence intensity was measured at ex/em 560/590 nm for the Alamar Blue assay. For the XTT assay, 200 µL of 20% 1 mg mL^{-1} XTT solution (Sigma-Aldrich) and 12.5 mM menadione (Sigma-

Aldrich) in DMEM were added and incubated for 3 h, and the conversion of tetrazolium salt XTT to a colored formazan derivative was measured at 450 nm. Non-treated controls were maintained throughout the cell viability assay. Blanks were subtracted from all test conditions. Six repeats were performed for each sample, and all results are shown as mean ± SD.

Minimum inhibitory concentration (MIC) and Minimal Bactericidal Concentration (MBC) assays

MIC was conducted using the broth dilution method.[2] Briefly, a single colony of the bacteria cultured on either blood or MHB II agar plates was suspended in MHB II broth and incubated at 37 °C with shaking to log phase. The suspended bacteria were then diluted with broth to reach a concentration of 10^6 CFU mL^{-1}. The polymers were diluted in 96-well plates using the double dilution method and the bacteria solution was added to the wells to reach a final concentration of 5×10^6 CFU mL^{-1}. Wells containing bacterial cells only and those containing polymers only were used as the positive and negative controls. The plates were incubated at 37 °C for 18 h and the optical density (OD = 620 nm) was used to determine the MIC values. For the MBC the same procedure was conducted, except that the plate counting method was used at the end to determine microbicidal concentrations. MIC and MBC tests were done in triplicate for each sample.

Antibacterial study of the cationic hydrogels

The antibacterial activity of the hydrogels were tested based on an established protocol[3] with modifications. Briefly, the cationic hydrogels (50 µL final volume) were prepared in the 96-well plates. The hydrogels were equilibrated with sterilized DI water for 20 min and surplus sterilized DI water was removed from the hydrogels. Bacterial solutions in log phase were diluted with MHB II broth to the desired concentrations ranging from 10^6 - 10^8 CFU mL^{-1}, and 25 µL bacterial solution was added above the hydrogels, and untreated 96-well plates were used as control. The 96-well plates were incubated at 37 °C for 6 h. After incubation the plate counting method was used to

calculate the killing percentages of the hydrogels towards different bacterial strains. Each sample was tested in triplicate.

In vitro cell infection assay

In vitro cell infection assays were conducted in 24-well cell culture plates (Costar) using antibiotic and serum-free medium. HaCaT cells were infected with 10^6 CFU mL^{-1} (MOI 5) of *S. aureus* 2569, MRSA, GAS, *E. coli* AB1 and *P. aeruginosa* AB2. The culture plates were centrifuged for 1 min at 350 g to enhance bacterial attachment. Two infection models were built. In the first, cells were infected with the bacterial suspension for 2 h, washed with PBS to remove non-adherent bacteria, and supplemented with fresh medium. Afterwards, H10K-G5 (30 µL final volume) was added. In the second model, cells were infected with bacterial suspensions and H10K-G5 was added at the same time. In both cases, the hydrogels were present in the medium throughout the entire infection period. Bacterial counts were assessed after a total 6 h post-infection. Bacterial suspensions were serially diluted and plated on blood agar plates. Total bacterial numbers were evaluated in relation to the number of bacteria added from the same experiment and normalized with respect to untreated infected control. Six repeats were done for each sample, and all results are shown as mean ± SD.

Total RNA extraction and real-time PCR analysis

HaCaT cells were treated with H10K-G5 (hydrogels were formed with 30 µL precursor solution) in DMEM for 6 h. Total RNA of HaCaT cells was determined via the RNeasy Mini kit (Qiagen) and transcribed to cDNA using the High-Capacity cDNA Reverse Transcription Kit (Applied Biosystems) according to the manufacturer's protocol. Expression of *RNASE7*, forward: 5’-GGA GTC ACA GCA CGA AGA CCA-3’, reverse: 5’-CAT GGC TGA GTT GCA TGC TTG A-3’) and *S100A7*, forward: 5’-GGC TAT GTC TCCCAG CAA -3’, reverse: 5’-CAC CAG ACG TGA TGA CAA -3’), *IL1B*, forward: 5’- CAC GAT GCA CCT GTA CGA TCA -3’, reverse: 5’- GTT GCT

CCA TAT CCT GTC CCT -3’) was analyzed using SYBR Green reagent in Rotor-Gene PCR cycler (Corbett Life Science). Human beta-actin (*ACTB*, forward: 5′-AAG AGA GGC ATC CTC ACC CT-3′ and reverse: 5′- TAC ATC GCT GGG GTG TTG-3′) was used as housekeeping gene. Relative expressions of target genes were presented as $2^{-\Delta CT}$ and fold change as $2^{-\Delta\Delta CT}$ compared to uninfected or non-treated control.

Immunofluorescence microscopy

For expression analysis of psoriasin and RNase7, sub-confluent HaCaT cells were treated with H10K-G5 (hydrogels were formed with 30 µL precursor solution) in DMEM for 24 h and fixed with 4% paraformaldehyde. Cells were stained with anti-psoriasin antibody (Santa Cruz Biotechnology), anti-RNase7 antibody (Novus Biologicals) at 1:200 dilution followed by the respective Alexa Fluor 647 and Alexa Fluor 488 conjugated secondary antibody (Life Technologies) at 1:400 dilution and counter-stained using 2.5 µg mL^{-1} 4′,6-diamidino-2-phenylindole (DAPI). Confocal images were acquired on a LSM 700 microscope (Carl Zeiss) using 63× oil immersion objective. All images were processed for intensity quantification by ImageJ software (NIH).

Free radical formation assay

HaCaT cells infected with *S. aureus 2569* (MOI 10) were treated with H10K-G5 (hydrogels were formed with 30 µL precursor solution) for 4 h. Supernatants were collected and mixed with equal volumes of Griess reagent (Cayman’s chemical) based on the manufacturer’s protocol. Optical density was measured at 550 nm, and free nitrite was evaluated and normalized to control infected cells. For total ROS analysis after 4 h infection, 10 µM DCFH-DA (a lipid permeable non-fluorescent dye) was added to the cells and they were incubated at 37 °C and 5% CO_2 for 2 h. Fluorescent intensity was measured at excitation 485 nm and emission 530 nm. Similarly, for mitochondrial ROS analysis after 4 h infection, cells were first washed with 1× Hanks' Balanced

Salt Solution (HBSS), then 5 µM Mitosox (Life Technologies) was added and left for 30 mins, then live cell imaging was done to measure the mitochondrial ROS. Fluorescent intensity was quantified using ImageJ software.

Enzyme-linked immunosorbent assay (ELISA)

HaCaT cells were treated with H10K-G5 (hydrogels were formed with 30 µL precursor solution) in DMEM for 24 h. Supernatants were collected and centrifuged at 350 g for 10 min and stored at -80 °C until needed. IL-1β (R&D Biosystems) ELISA was analyzed according to the manufacturer's recommendations. Untreated cells served as control.

Inhibition of biofilm formation

E. coli #12 and *E. coli* WE1*bcsA* (curli +/ cellulose -) strains were used in this study.[4] H10K-G5 and bacteria were added at the same time and control wells were only added with bacteria in a 24-well plate. *E. coli* at a concentration of 5 x 10^6 CFU mL^{-1} were grown for 72 h in LB broth at 37 °C without shaking. The biofilm was washed with 1× PBS and stained with 550 µL of crystal violet (0.3%) for 10 min at room temperature. Any unstained dye was removed, and the stained biofilms were washed with water. The dye was solubilized with 600 µL of 80% ethanol and 20% acetone, and the OD was measured at 570 nm.

Supplementary Results

Table S1. MIC values of hydroxy-terminated HBDLDs and β-alanine.

		Bacteria				
		E. coli 178	*E. coli* 208	*S. aureus* 2569	*S. aureus* 7920	*P. aeruginosa* 22644
		μg mL^{-1}	μg mL^{-1}	μg mL^{-1}	μg mL^{-1}	μg mL^{-1}
	Polymers					
MIC values	PEG6K-G5-OH	>2000	>2000	>2000	>2000	>2000
	PEG6 K-G6-OH	>2000	>2000	>2000	>2000	>2000
	PEG10K-G5-OH	>2000	>2000	>2000	>2000	>2000
	PEG10K-G6-OH	>2000	>2000	>2000	>2000	>2000
	PEG20K-G5-OH	>2000	>2000	>2000	>2000	>2000
	β-alanine	>2000	>2000	>2000	>2000	>2000

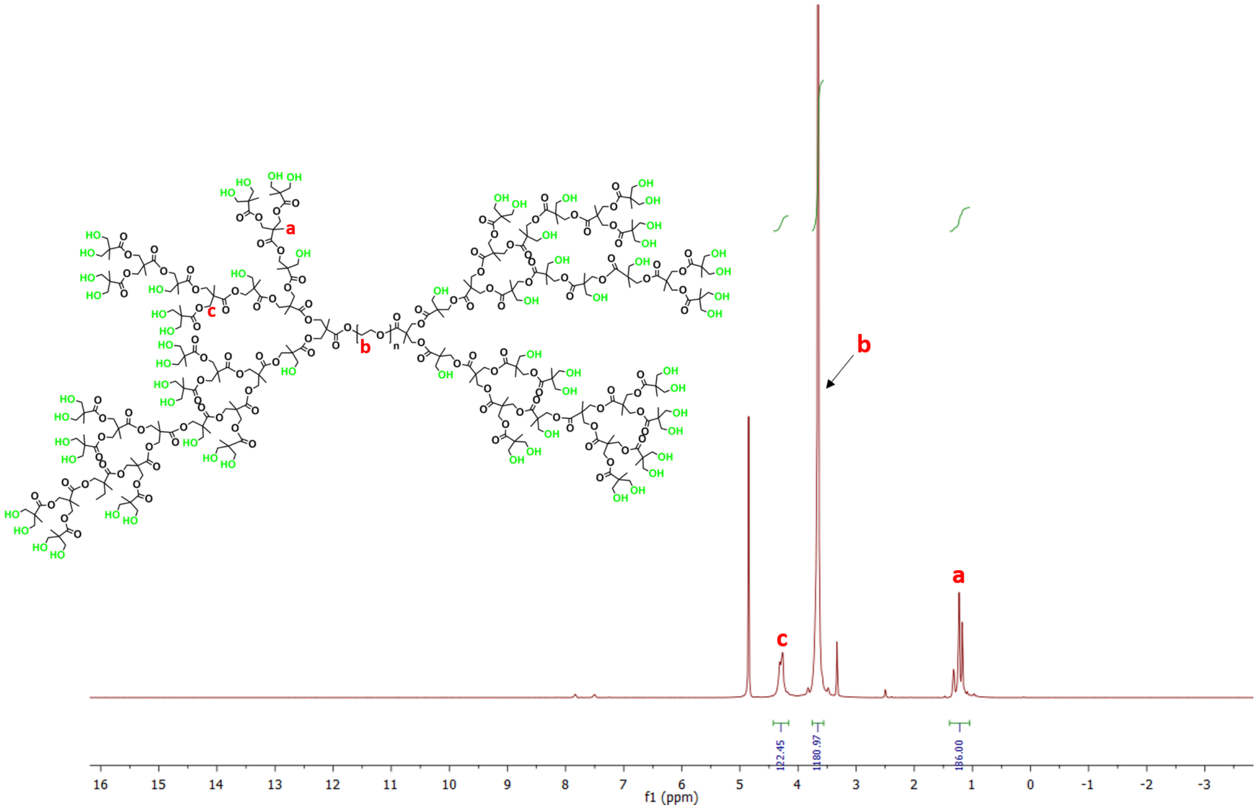

Figure S1. ^{1}H NMR spectroscopy spectrum of PEG10K-G5-OH in MeOD to confirm the full generation of the copolymer is reached. The calculated actual ratio of protons from the methyl groups of bis-MPA (a), PEG (b), and internal methylene group of Bis-MPA (c) is 186:1180.97:122.45, which is very close to the theoretical ratio of 186: 909:124.

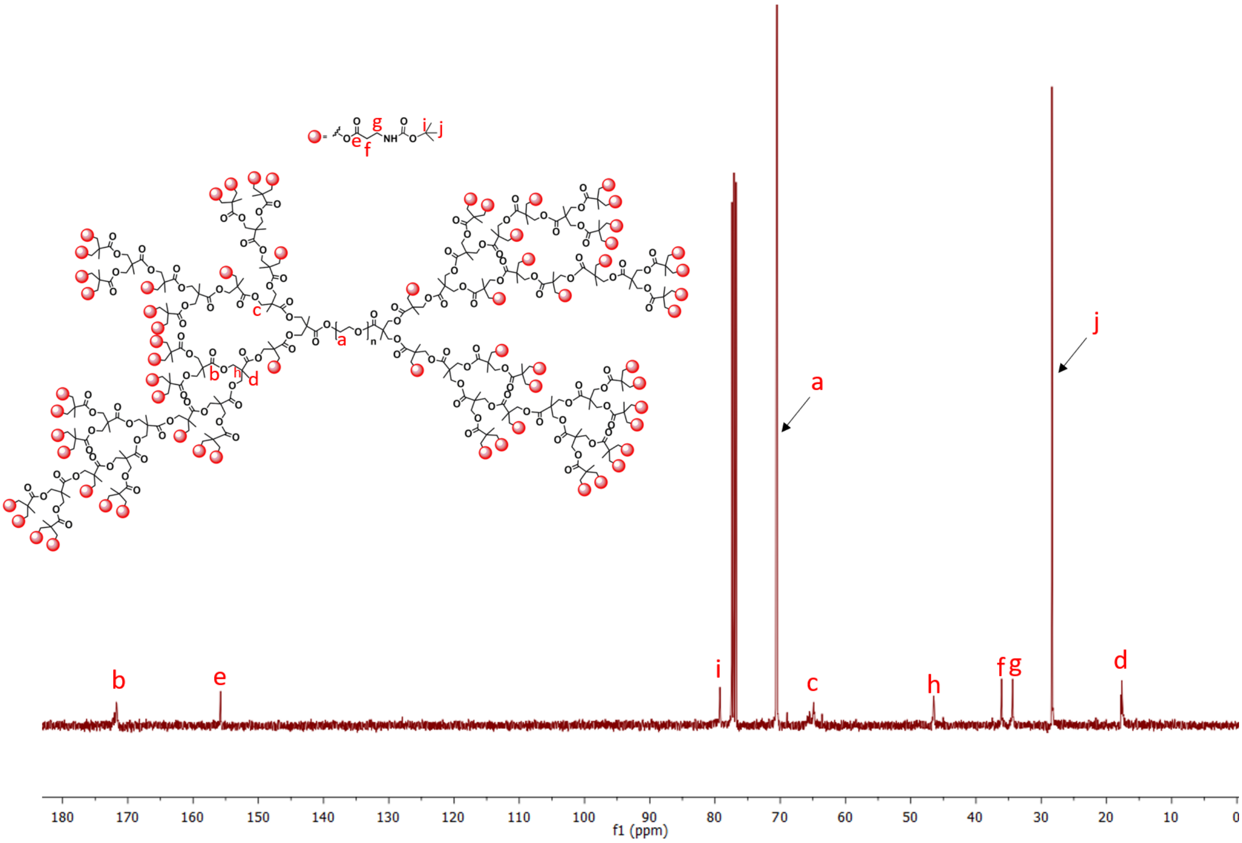

Figure S2. ^{13}C NMR spectroscopy spectrum of PEG10K-G5-NH-BOC in $CDCl_3$ to confirm the full substitution of hydroxy groups. There is no peak at around 50.2 ppm (-COO-**C**-($(CH_2$-$OH)_2$, bis-MPA at the terminal), and peak f (at 36.08 ppm) and peak g (at 34.38 ppm) corresponding to β-alanine appeared in the ^{13}C NMR spectroscopy, indicating that all hydroxy groups at the terminal positions were substituted.

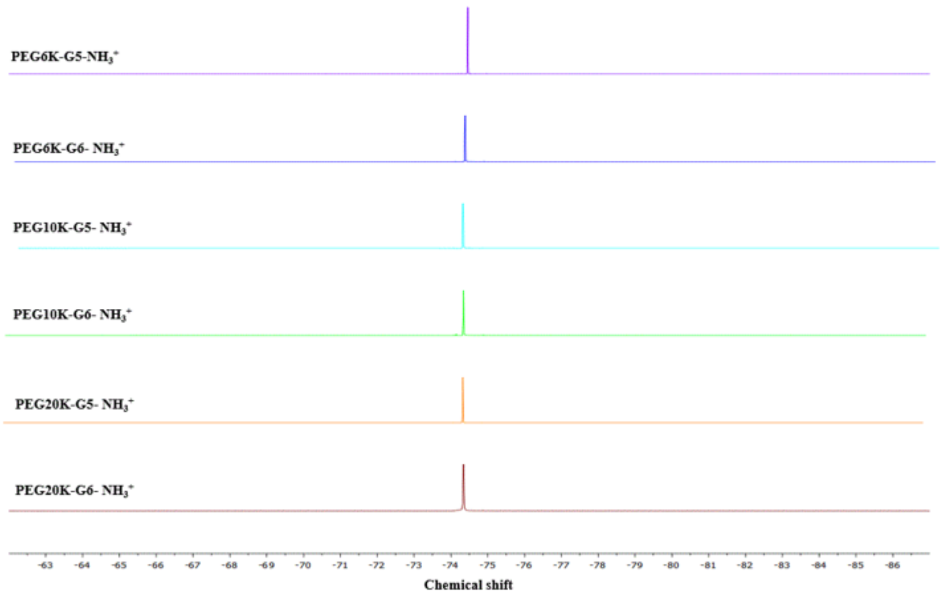

Figure S3. ^{19}F NMR spectroscopy spectra of the amino-functional HBDLDs.

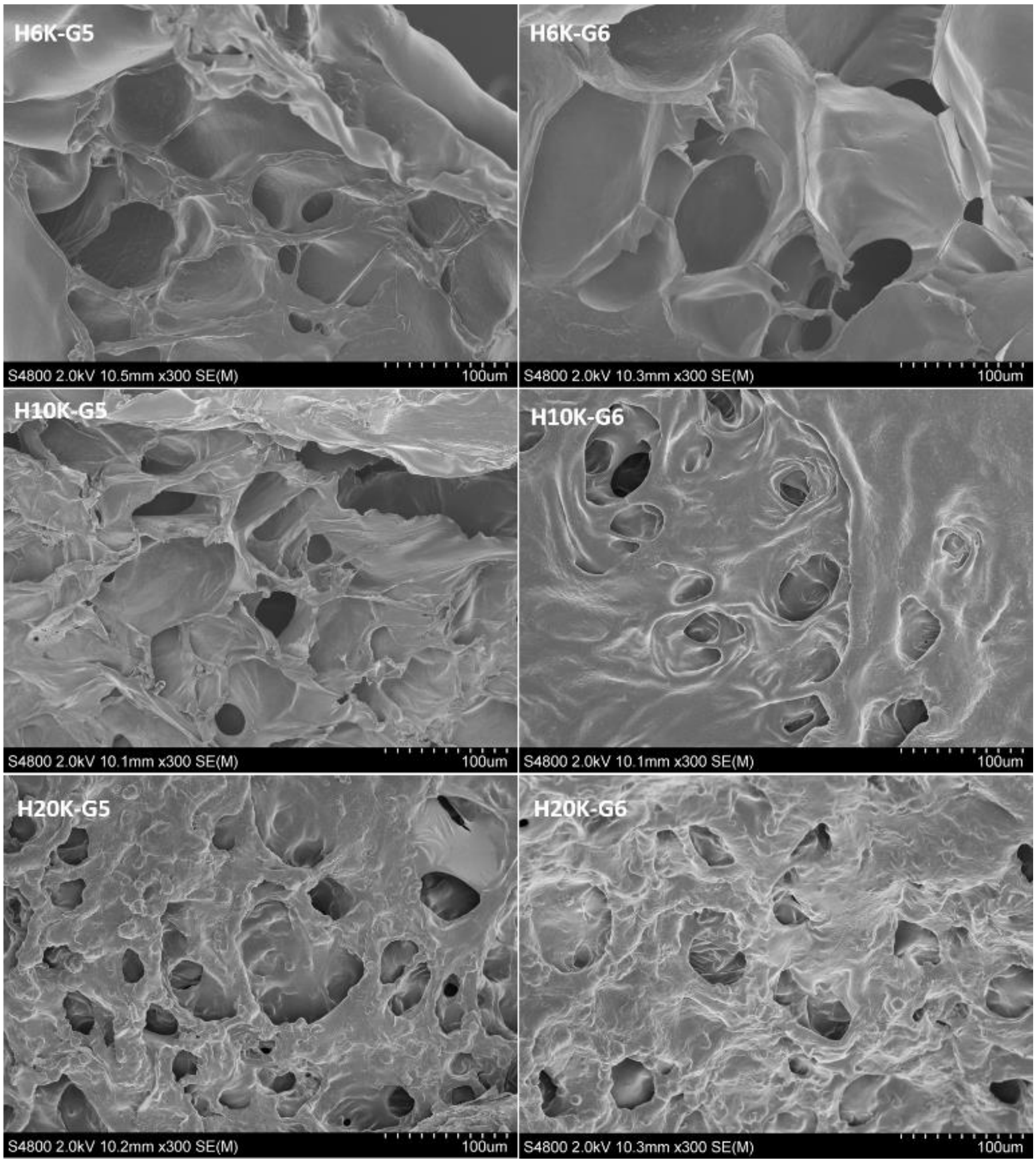

Figure S4. Porous structure of amino-functional hydrogels H6K-G5, H6K-G6, H10K-G5, H10K-G6, H20K-G5 and H20K-G6.

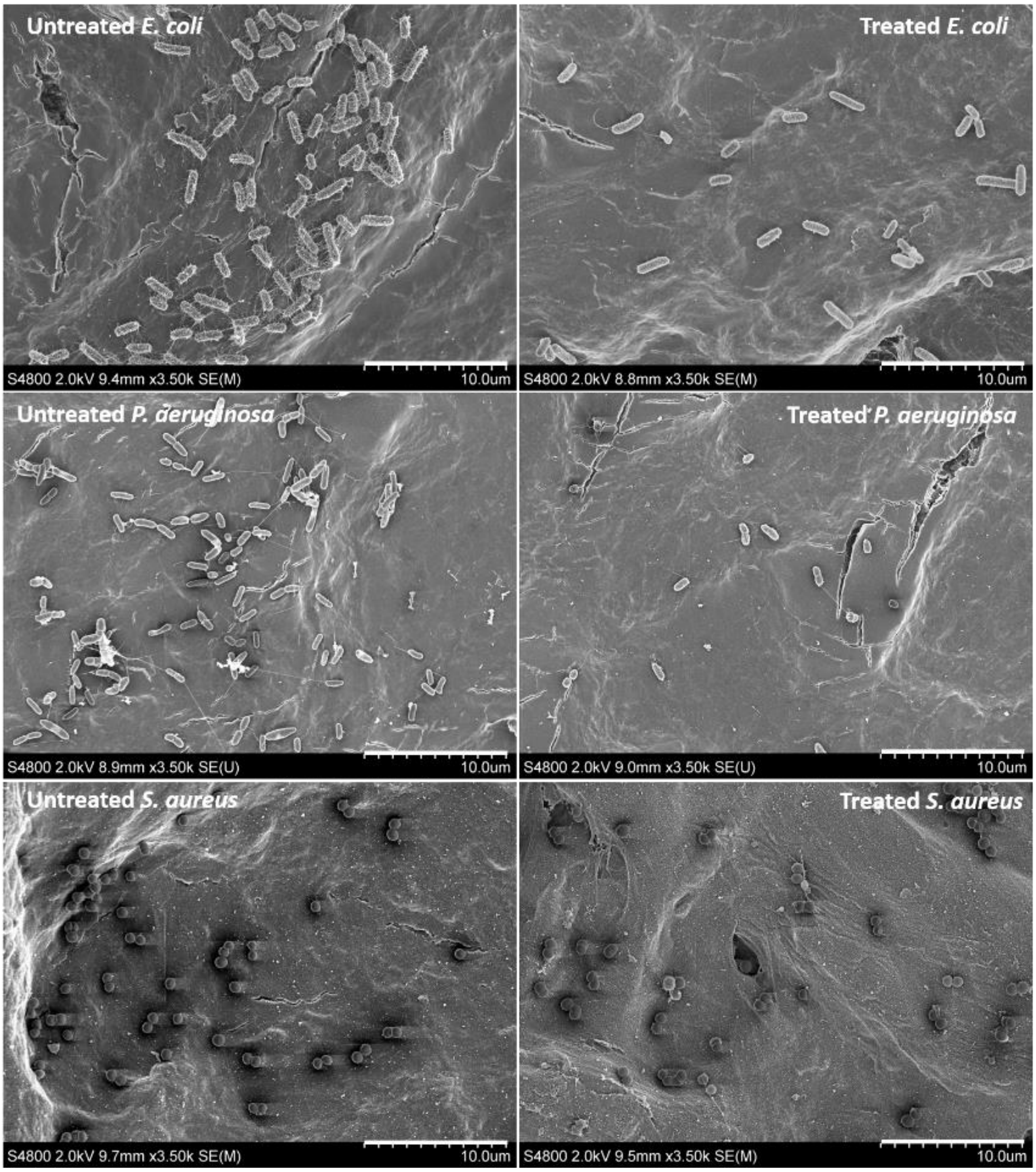

Figure S5. SEM images showing the density of the attached bacteria on the filter paper, less bacterial attachment after the treatment with 250 μg mL^{-1} PEG10K-G5-NH_3^+ for 6 h at 37 °C.

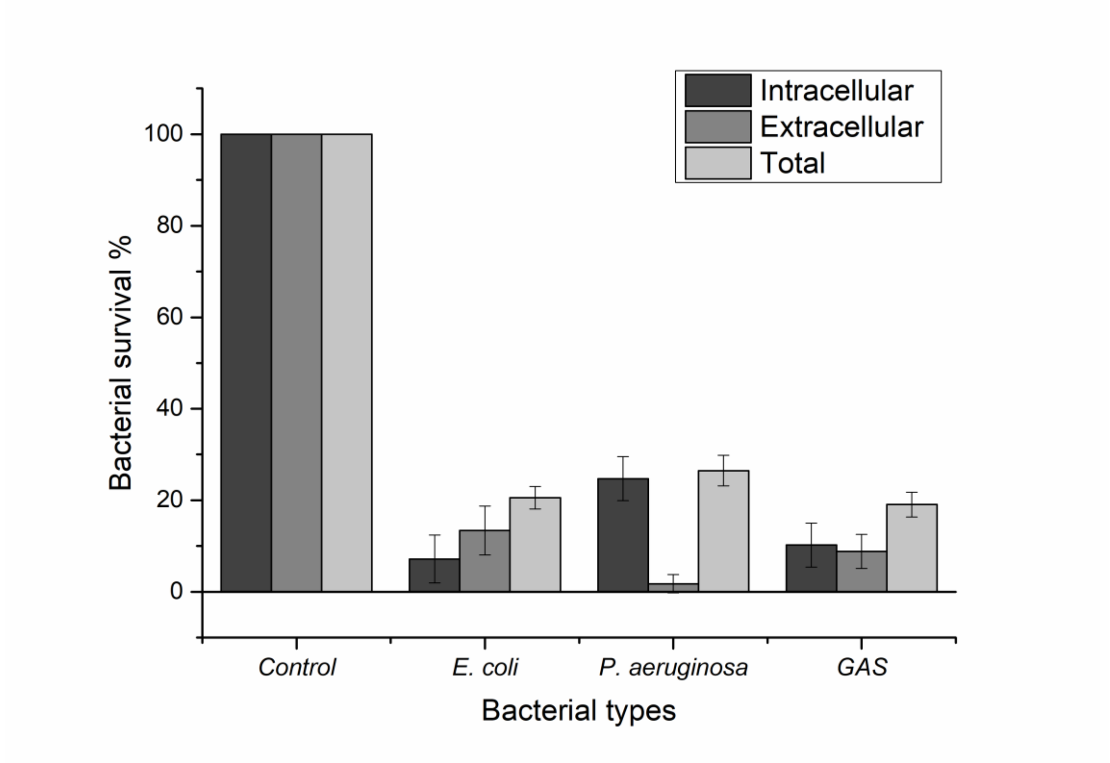

Figure S6. *In vitro* cell infection assays using HaCaT keratinocytes against *E. coli* AB1, *P. aeruginosa* AB2 and Group A Streptococcus (GAS) under the treatment of H10K-G5. All data is shown as a mean value ± SD (n = 6).

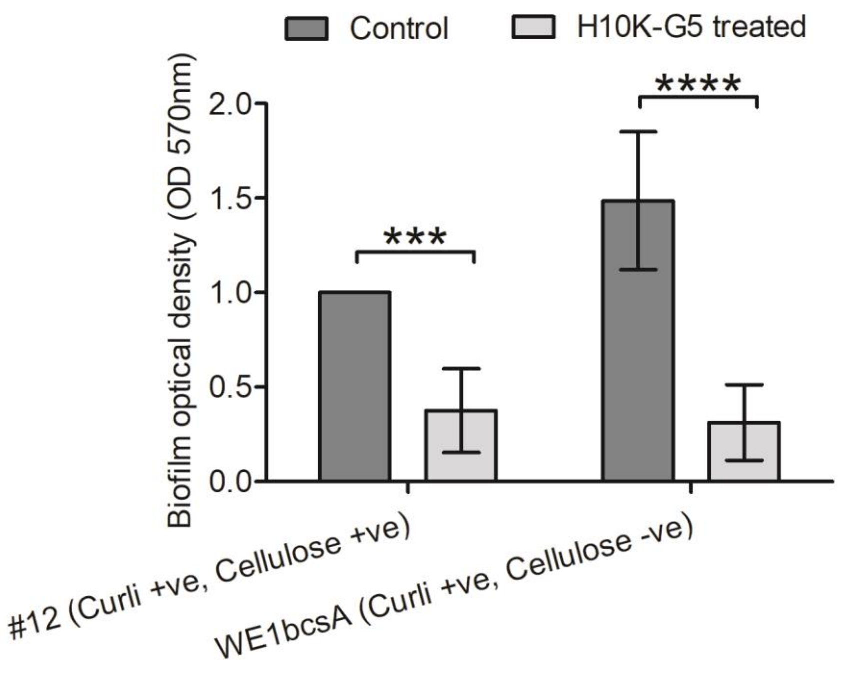

Figure S7. Inhibition of initial *E. coli* biofilm formation by the cationic hydrogels determined by the crystal violet assay. All data are shown as a mean value ± SD (n = 6). Significance levels mentioned as ***P < 0.001 and ****P < 0.0001.

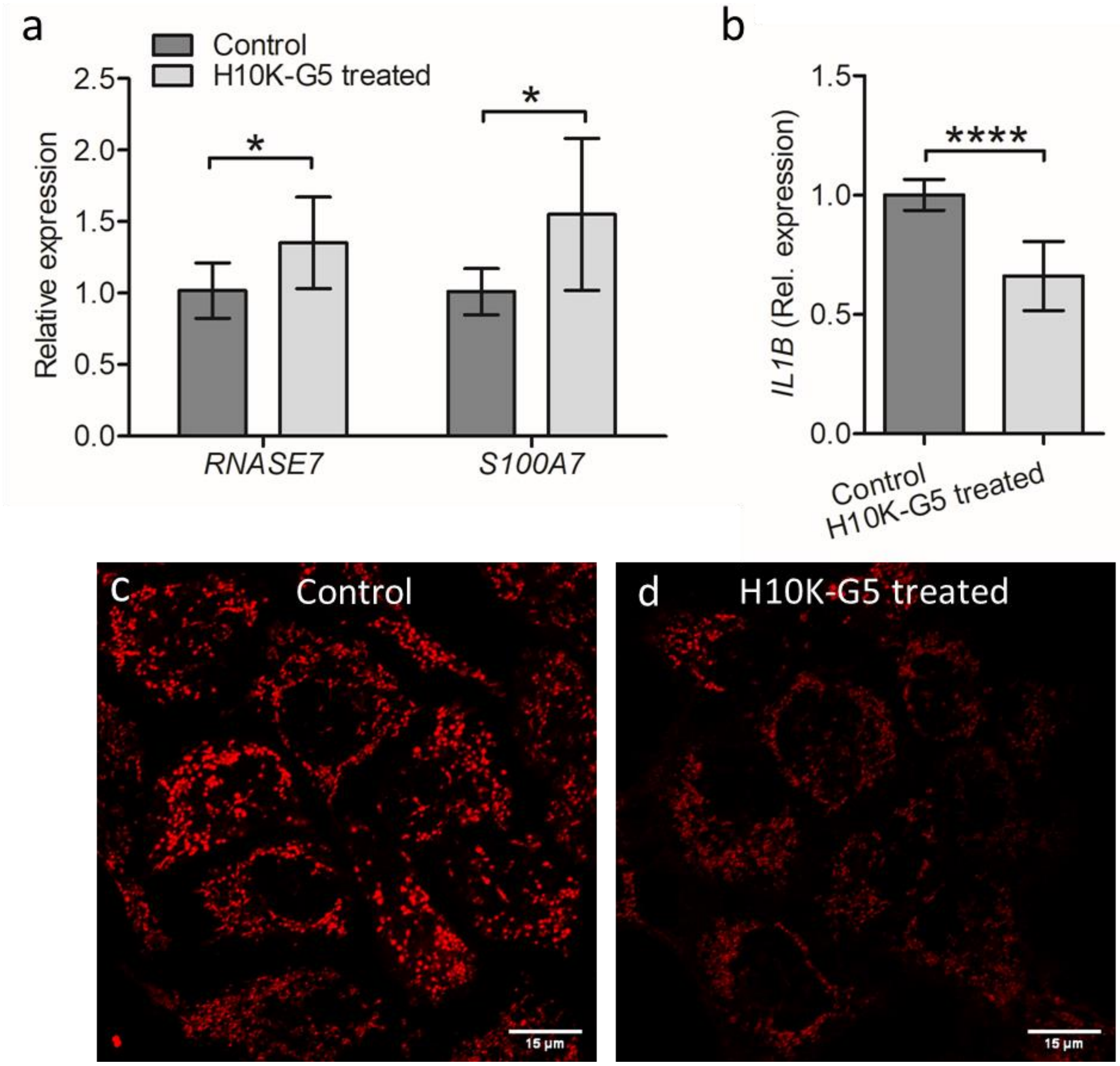

Figure S8. (a) Expression of antimicrobial peptides *RNASE7* and *S100A7* mRNA in HaCaT cells (n=4). (b) Relative expression of *IL1B* mRNA in HaCaT cells (n=4). (c, d) Representative image of mitochondrial ROS levels in *S. aureus*-infected HaCaT cells (n=3). Significance levels mentioned as *P < 0.05 and ****P < 0.0001.

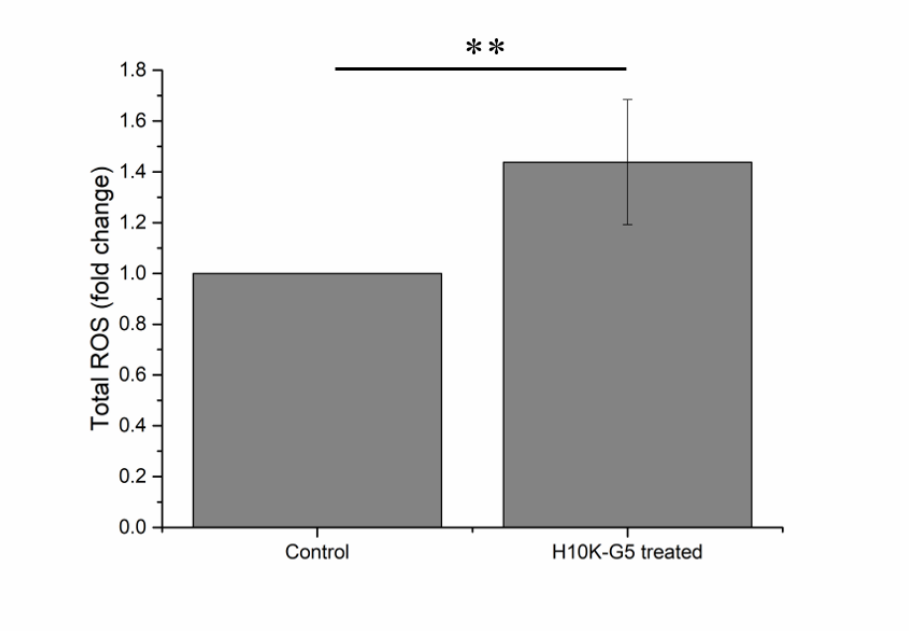

Figure S9. Total ROS of *S. aureus*-infected HaCaT cells with and without treatment with H10K-G5. All data is shown as a mean value ± SD (n = 6). Significance levels mentioned as **$P < 0.01$.

References

[1] O. C. J. Andrén, M. V. Walter, T. Yang, A. Hult, M. Malkoch, *Macromolecules* **2013**, 46, 3726.
[2] I. Wiegand, K. Hilpert, R. E. Hancock, *Nature protocols* **2008**, 3, 163.
[3] D. A. Salick, J. K. Kretsinger, D. J. Pochan, J. P. Schneider, *Journal of the American Chemical Society* **2007**, 129, 14793.
[4] Y. Kai-Larsen, P. Lüthje, M. Chromek, V. Peters, X. Wang, Å. Holm, L. Kádas, K.-O. Hedlund, J. Johansson, M. R. Chapman, *PLoS Pathog* **2010**, 6, e1001010.
